# Differential Morphological Diagnosis of Various Forms of Congenital Hyperinsulinism in Children

**DOI:** 10.3389/fendo.2021.710947

**Published:** 2021-08-23

**Authors:** Lubov Borisovna Mitrofanova, Anastasia Arkadyevna Perminova, Daria Viktorovna Ryzhkova, Anna Andreyevna Sukhotskaya, Vladimir Gireyevich Bairov, Irina Leorovna Nikitina

**Affiliations:** Almazov National Medical Research Center, St. Petersburg, Russia

**Keywords:** forms of congenital hyperinsulinism, immunohistochemistry, cytological analysis, intraoperative differential diagnosis, *[18F]-DOPA PET/CT imaging*

## Abstract

**Introduction:**

Congenital hyperinsulinism (CHI) has diffuse (CHI-D), focal (CHI-F) and atypical (CHI-A) forms. Surgical management depends on preoperative [18F]-DOPA PET/CT and intraoperative morphological differential diagnosis of CHI forms. Objective: to improve differential diagnosis of CHI forms by comparative analysis [18F]-DOPA PET/CT data, as well as cytological, histological and immunohistochemical analysis (CHIA).

**Materials and Methods:**

The study included 35 CHI patients aged 3.2 ± 2.0 months; 10 patients who died from congenital heart disease at the age of 3.2 ± 2.9 months (control group). We used PET/CT, CHIA of pancreas with antibodies to ChrA, insulin, Isl1, Nkx2.2, SST, NeuroD1, SSTR2, SSTR5, DR1, DR2, DR5; fluorescence microscopy with NeuroD1/ChrA, Isl1/insulin, insulin/SSTR2, DR2/NeuroD1 cocktails.

**Results:**

Intraoperative examination of pancreatic smears showed the presence of large nuclei, on average, in: 14.5 ± 3.5 cells of CHI-F; 8.4 ± 1.1 of CHI-D; and 4.5 ± 0.7 of control group (from 10 fields of view, x400). The percentage of Isl1+ and NeuroD1+endocrinocytes significantly differed from that in the control for all forms of CHI. The percentage of NeuroD1+exocrinocytes was also significantly higher than in the control. The proportion of ChrA+ and DR2+endocrinocytes was higher in CHI-D than in CHI-F, while the proportion of insulin+cells was higher in CHI-A. The number of SST+cells was significantly higher in CHI-D and CHI-F than in CHI-A.

**Conclusion:**

For intraoperative differential diagnosis of CHI forms, in addition to frozen sections, quantitative cytological analysis can be used. In quantitative immunohistochemistry, CHI forms differ in the expression of ChrA, insulin, SST and DR2. The development of a NeuroD1 inhibitor would be advisable for targeted therapy of CHI.

## Introduction

Congenital hyperinsulinism (CHI) is a group of disorders that cause persistent hypoglycemia due to congenital excess secretion of insulin; it does not include acquired conditions such as insulinoma, iatrogenic hyperinsulinemia, or dumping syndrome. It is the most common cause of persistent hypoglycemia in newborns and infants (1, 2). Mutations in 12 key genes (ABCC8, KCNJ11, GLUD1, GCK, HADH, SLC16A1, UCP2, HNF4A, HNF1A, HK1, PGM1, PMM2), which are involved in regulation of β-cell insulin secretion, can lead to CHI (3–5).

There are 3 main histological forms of CHI: focal (CHI-F); diffuse (CHI-D); and atypical (CHI-A) (6). CHI-F represents 40-50% of cases and is defined as a limited area of the pancreas with adenomatous β-cell hyperplasia, which leads to fusion of the islets of Langerhans. Large islets are generally limited to only a few lobes. This form has the potential to be cured with partial pancreatectomy, but islets are difficult to recognize on frozen sections in urgent biopsy. CHI-D includes islets of Langerhans throughout the pancreas and is histologically characterized by hypertrophy of multiple β-cell nuclei in most islets of Langerhans. In CHI-A, 2 types of islets coexist: large islets with β-cells rich in cytoplasm and sometimes enlarged nuclei; and small islets with β-cells featuring small cytoplasm and small nuclei. The changes are characterized by morphological mosaicism. Pathologists must recognize this mosaicism in intraoperative frozen sections because the extent of the operation depends on it. Insulinoma is characterized by a loss of normal pancreas architecture due to localized hyperplasia of insulin-producing cells. An insulinoma may have a limited number of other endocrine or exocrine cells. In contrast, CHI-F maintains islet and lobular architecture involving glucagon and other non-β-endocrine cells in the lesion. Insulinoma may present sporadically as a solitary adenoma or as part of multiple endocrine neoplasia in older children.

Diagnosis of CHI is based on clinical, laboratory, nuclear medicine, molecular genetic, and histopathological parameters. Positron emission tomography (PET) with [18F] -fluoro-L-dihydroxyphenylalanine ([18F] -DOPA) has been successfully used for differential diagnosis of CHI morphological forms. A precursor of catecholamines, L-dihydroxyphenylalanine (L-DOPA), is converted to dopamine by aromatic amino acid decarboxylase (AADC) in beta cells. The radiotracer [18F] -fluoro-L-dihydroxyphenylalanine ([18F] -DOPA) concentrates in beta cells of the pancreas. The pattern of [18F] -DOPA distribution in the pancreas is associated with focal or diffuse hypermetabolism of dopamine in the pancreatic islets and correlated with the immunohistochemical expression of AADC, proinsulin and insulin. Hybrid [18F] -DOPA PET/CT or PET/MRI (7–9) permits precise localization of a focus of adenomatosis before surgery.

Chromogranin A is a member of the granin glycoprotein family, which is expressed by endocrine and neuroendocrine cells of various organs. Intracellularly, chromogranin A contributes to regulation of secretion and produces several cleavage products. Some of its cleavage products modulate the function of hormones in an autocrine and paracrine manner. According to the available literature, chromogranin A contributes to the pathogenesis of diabetes mellitus (10).

The Isl1 transcription factor is required to stimulate the proliferation of pancreatic islet cells and their “survival” in the embryonic and postnatal periods (11, 12). The mechanism of its interaction with other transcription factors in CHI is poorly understood.

NeuroD1 is a transcription factor that was found in the adult nervous system and in the endocrine cells of primitive neuroectodermal tumors (13). It is known that this factor is involved in neurogenesis, including controlling the possibility of transdifferentiation of other types of cells into neurons; it is also a regulator of brain development as a whole (14). NeuroD1 is expressed in the pituitary gland and in progenitor cells of the endocrine pancreas, both at the stages of its formation and in adults (15, 16). It is also known that, in addition to α and β cells, NeuroD1 differentiates PP and δ cells, activates the glucagon promoter, and suppresses somatostatin expression (17).

The transcription factor Nkx2.2, like NeuroD1, is involved both in the early development of the central nervous system and in differentiation of α, β, PP, and δ islet cell lineages of the endocrine function of the pancreas (18). It is of decisive importance in the differentiation of pancreatic endocrine cells in the developing mouse embryo (19). Depending on context, it can repress or activate NeuroD1 (20).

Somatostatin (SST) is a peptide hormone that is secreted by delta cells (δ cells) of the pancreas. It binds to G-protein coupled SST receptors to regulate a variety of selective and local specific functions, such as hormone inhibition, neurotransmission, and cell proliferation. SST plays an important role in regulation of both insulin and glucagon secretion, in response to changes in glucose level, through a negative feedback mechanism (21, 22). The half-life of SST is only 1-3 minutes due to rapid degradation by peptidases in plasma and tissues. To achieve a therapeutic effect, direct continuous intravenous or subcutaneous infusion is required. These limitations prompted the development of SST analogs, such as octreotide and lanreotide, which have longer half-lives and therefore can be injected. SST analogs are used to treat various forms of CHI, and the therapeutic effect is achieved by suppressing insulin secretion by β-cells of the pancreas through complex mechanisms.

*In vitro* studies show that dopamine inhibits glucose-stimulated insulin secretion in the pancreas (23). It has been suggested that this may be due to suppression of D2-like receptors, which causes: increased release of catecholamines; and decreased cell membrane depolarization leading to decreased calcium influx into the cytosol and reduced insulin secretion (24). Disruption of D2 receptors leads to impaired insulin secretion by the pancreas and causes glucose intolerance (25). Thus, dopamine receptors expressed in pancreatic beta cells modulate insulin release.

Objective: to improve differential diagnosis of various forms of CHI by comparative analysis of pediatric pancreatic samples using [18F]-DOPA PET/CT imaging, as well as cytological, histological and immunohistochemical analysis.

## Materials and Methods

The work was carried out at the Almazov National Medical Research Centre. The study was performed in accordance with the principles of the Declaration of Helsinki and approved by the local ethics committee at the Almazov NMRС. The study included 35 patients (12 boys, 23 girls) diagnosed with CHI, aged from 1 month to 4 years. The average age of the examined patients was 3.2 ± 2.0 months. The control group samples included autopsy material (normal pancreatic body specimen) of 10 patients who died of cardiovascular defects. The average age of control group patients was 3.2 ± 2.9 months, consisting of 4 girls and 6 boys.

All children with CHI underwent: pancreatic [18F] fluoro-l-dihydroxyphenylalanine ([18F]-DOPA) integrated positron emission tomography and computed tomography (PET/CT); genetic study; and surgery with morphological examination of frozen sections and paraffin-embedded pancreatic preparations. According to the anamnesis (**Table 1**), in 48.6% of children with CHI, the birth weight was more than 4000 g. Clinical manifestations of congenital hyperinsulinism in the form of hypoglycemia were observed in almost all children, regardless of the morphological form of the disease, already within the 1st week of life. Convulsive syndrome was observed in 68.6% of patients (24 patients). In 23 children, mutations were located in the ABCC8 and/or KCNJ11 genes encoding ATP-dependent potassium channel proteins. Beckwith-Widemann syndrome was ruled out in all examined children due to an absence of corresponding clinical signs. Changes on chromosome 11p15 were not detected in any child. In addition, a genetic study was performed with 16 pairs of parents, and one mother, of the patients.

**Table 1:** Patient clinical and morphological characteristics.

№	Sex	Age, months	Birth weight, grams	Convulsions	Mutations in patients	Mutations in parents of patients	PET CT/SUV ratio	Surgery volume (pancreas)	Morphological form	Outcome
1	m	4	3260	No	none	no data	focal/1.87	subtotal head and body resection	focal	complete recovery, no therapy
2	f	5	4700	Yes	ABCC8 heterozygous	ABCC8 heterozygous in father	focal/3.59	subtotal head resection	focal	complete recovery, no therapy
3	f	5	3700	Yes	ABCC8 heterozygous	ABCC8 heterozygous in father	focal/2.84	subtotal head and body resection	focal	complete recovery, no therapy
4	m	4	3420	Yes	KCNJ11 heterozygous	KCNJ11 heterozygous in father	focal/1.90	subtotal head resection	focal	complete recovery, no therapy
5	m	2	4530	Yes	ABCC8 heterozygous and KCNJ11 heterozygous	KCNJ11 heterozygous in father	focal/1.90	body resection	focal	complete recovery, no therapy
6	m	5	3300	Yes	ABCC8 heterozygous	ABCC8 heterozygous in father	focal/1.87	subtotal head resection	focal	complete recovery, no therapy
7	f	2	4000	Yes	ABCC8 heterozygous and KCNJ11 heterozygous	no data	focal/2.58	subtotal head resection	focal	complete recovery, no therapy
8	f	7	4060	Yes	ABCC8 heterozygous	ABCC8 heterozygous in father	focal/1.59	tail and body resection	focal	complete recovery, no therapy
9	m	5	3600	Yes	ABCC8 heterozygous	ABCC8 heterozygous in father	focal/1.42	subtotal pancreatectomy	focal	complete recovery, no therapy
10	m	8	2950	Yes	no data	no data	focal/2.06	distal resection	focal	complete recovery, no therapy
11	m	3	4040	Yes	ABCC8 heterozygous	ABCC8 heterozygous in father	focal/2.08	subtotal head resection	focal	complete recovery, no therapy
12	m	2	4400	Yes	ABCC8 heterozygous	ABCC8 heterozygous in father	focal/2.16	body resection	focal	complete recovery, no therapy
13	f	3	4170	No	ABCC8 heterozygous	ABCC8 heterozygous in father	focal/2.32	subtotal head and body resection	focal	complete recovery, no therapy
14	f	6	4740	Yes	no data	no data	focal/1.53	subtotal head and body resection	focal	complete recovery, no therapy
15	f	2	3750	Yes	ABCC8 heterozygous	ABCC8 heterozygous in father	focal1.85	body resection	focal	complete recovery, no therapy
16	m	3	3350	No	ABCC8 heterozygous	ABCC8 heterozygous in father	focal/1.75	subtotal head resection	focal	complete recovery, no therapy
17	f	1	3610	Yes	ABCC8 heterozygous	ABCC8 heterozygous in father	focal/1.50	subtotal head and body resection	focal	complete recovery, no therapy
18	f	2	3270	Yes	no data	no data	focal/1.68	subtotal head and body resection	focal	complete recovery, no therapy
19	m	2	3980	Yes	no data	no data	focal/2.25	subtotal head resection	focal	complete recovery, no therapy
20	f	2	3740	Yes	no data	no data	diffuse/1.15	subtotal pancreatectomy	diffuse	diabetes
21	f	2	3600	Yes	HNF4A heterozygous	no data	diffuse or focal/1.42	subtotal pancreatectomy	diffuse	diabetes
22	f	1	3400	Yes	none	none	no data	subtotal pancreatectomy	diffuse	relapse, on insulinostatic therapy
23	f	3	5550	Yes	ABCC8 homozygous	ABCC8 heterozygous in father and mother	diffuse/1.02	subtotal pancreatectomy	diffuse	no therapy
24	f	33	3430	Yes	compound heterozygosity for 2 mutations in the ABCC8 gene	ABCC8 heterozygous in father and mother	diffuse/1.14	subtotal pancreatectomy	diffuse	relapse, on insulinostatic therapy
25	f	1	4750	Yes	ABCC8 homozygous	no data	diffuse/1.01	subtotal pancreatectomy	diffuse	diabetes
26	m	1	4300	No	no mutations in ABCC8 or KCNJ11	no data	diffuse/1.11	subtotal pancreatectomy	diffuse	diabetes
27	f	2	4380	No	KCNJ11 homozygous	no data	diffuse/1.23	subtotal pancreatectomy	diffuse	no therapy
28	f	58	5130	Yes	no mutations in ABCC8 or KCNJ11	no data	diffuse/1.17	subtotal pancreatectomy	diffuse	relapse, on insulinostatic therapy
29	f	1	4060	Yes	compound heterozygosity for 2 mutations in the ABCC8 gene	no data	diffuse/1.16	subtotal pancreatectomy	diffuse	diabetes
30	f	1,5	3980	Yes	ABCC8 heterozygous	no data	diffuse/1.36	subtotal pancreatectomy	diffuse	no therapy
31	f	3	4300	Yes	ABCC8 heterozygous	no data	focal/2.97	subtotal head resection	atypical	diabetes
32	f	7	2260	Yes	KCNJ11 homozygous and HNF4A heterozygous	no data	focal/1.87	subtotal pancreatectomy	atypical	diabetes
33	m	5	4220	No	ABCC8 heterozygous	no mutations in mother	multifocal/1.54	tail and body resection	atypical	no therapy
34	f	26	3060	No	no data	no data	multifocal or atypical/2.07	subtotal pancreatectomy	atypical	no therapy
35	f	16	4280	No	PMM2 heterozygous	no data	multifocal/1.55	subtotal head and body resection	atypical	relapse, on insulinostatic therapy

PET/CT scans were performed at the Almazov National Medical Research Centre PET facility. Medications that might affect pancreatic β-cell function (diazoxide, octreotide, glucagon) were discontinued 5 days, 2 days, or 12 hours prior to PET/CT scan, respectively. Patients were sedated during PET/CT procedures. Low-dose CT scans were used for attenuation correction. PET acquisition began 10 min after injection of [18F]-DOPA at a radioactive dose of 4 MBq/kg body weight; four consecutive 10-min scans were performed. PET scans were assessed visually and semiquantitatively. On the PET images (obtained 50-60 minutes after injection), three regions of interest (ROI) were drawn over the head, body, and tail of the pancreas to measurement maximal standardized uptake values (SUVmax). The form of CHI was determined according to pancreatic index as a ratio between SUVmax in the most active part of pancreas and SUVmax in the second most active part of pancreas (26). Using a threshold value with PET/CT, the number of patients were categorized as follows: focal form in 21 patients; diffuse form in 14 patients, and multifocal form in 2 patients. Inconclusive PET results were obtained in 2 patients.

### Histological Examination

Histological diagnosis of CHI was made intraoperatively using light microscopy of hematoxylin and eosin stained smears and frozen sections of the pancreas. Morphological assessments were preliminary. Final conclusions about CHI form were made after immunohistochemical examination of pancreas paraffin sections.

### Immunohistochemical Study

Immunohistochemical analysis of pancreatic samples from 35 patients with CHI and 10 patients from the control group was carried out according to a standard method (27) using antibodies to: chromogranin A (rabbit polyclonal antibody, dilution 1:400, Diagnostic BioSystems, The Netherlands); insulin (mouse monoclonal antibody, clone K36aC10, 1:50 dilution, Diagnostic BioSystems, The Netherlands); Isl1 (rabbit polyclonal antibody, 1:1000 dilution, ThermoFisher, USA); Nkx 2.2 (rabbit monoclonal antibody, clone EPR14638, dilution 1:20, Abcam, UK); somatostatin (SST, rabbit polyclonal antibody, dilution 1:1000, Dako, Denmark); NeuroD1 (mouse monoclonal antibody, clone 3H8, dilution 1:1000, Novus Biologicals, USA); somatostatin receptor type 2 (SSTR2, rabbit monoclonal antibody, clone EP149, dilution 1:200, Abcam, UK); somatostatin receptor type 5 (SSTR5, rabbit monoclonal antibody, clone UMB4, dilution 1:100, Abcam, UK); type 1 dopamine receptor (DR1, rabbit polyclonal antibody, dilution 1:50, Novus Biologicals, USA); type 2 dopamine receptor (DR2, mouse monoclonal antibody, clone B10, dilution 1:100, Santa Cruz Biotechnology, USA); and type 5 dopamine receptor (DR5, rabbit polyclonal antibody, 1:100 dilution, ThermoFisher, USA). Micrographs were taken with a Leica DM4000 microscope and a Leica Aperio AT2 scanning microscope.

### Immunofluorescence Study

Immunofluorescence microscopy and confocal laser scanning microscopy, for patient 3 with CHI-F and patient 26 with CHI-D (see **Table 1**), were performed on pancreas paraffin sections with cocktails: NeuroD1/chromogranin A; Isl1/insulin; insulin/SSTR2; and DR2/NeuroD1 (antibodies and dilutions as described above). Alexa Fluor 488^®^ goat anti-rabbit (Abcam, UK, 1:1000) and Alexa Fluor 647^®^ goat anti-mouse (Abcam, UK) were used as secondary antibodies. Sections were counterstained with DAPI (AppliChem). In result, NeuroD1 and insulin signals were seen as green fluorescence. Chr A, Isl1, DR2, and SSTR2 signals were seen as red fluorescence. Contrasted nuclei were seen as blue fluorescence. Preparations were analyzed using a Leica DM6000B microscope and a Leica TCS SP8 confocal laser scanning microscope (Germany).

### Morphometry and Statistics

Morphometric analysis was performed using an automated image analyzer (Image Scope Color M, Russia). In order to analyze the relative quantities of cells with enlarged nuclei and cells expressing select antigens, 10 high-power fields (400x magnification) were evaluated per specimen. Percentages of the average number of expressing cells, in relation to overall cells, were separately calculated. Pancreas micrographs were made from 2 patients and analyzed (10 high power fields, 400x) for evaluation of the most prominent double staining combinations: NeuroD1/chromogranin А; Isl1/insulin; insulin/SSTR2; and DR2/NeuroD1 combinations. The same-cell antigen coexpression coefficient was defined as the ratio of double-stained cells to the total number of cells, expressed as percentage. Antigen coexpression coefficients were determined using image analysis software (Image Scope Color M, Russia).

Statistical analysis of the acquired data was done using Statistica v.10 software (StatSoft, Russia). For normal distributions, the significance of differences in quantitative characteristics was interpreted using the Student’s t-test. For other types of distribution, we used non-parametric methods of analysis, namely the Mann-Whitney test for independent samples and the Wilcoxon test. Differences between groups were defined as significant when p<0.05.

In order to evaluate the correlation of two variables, we applied Spearman rank correlation analysis. Correlation coefficient (r) interpretation: r <0.3 as weak association; r=0.3-0.5 as moderate; r= 0.5-0.7 as significant; r=0.7-0.9 as strong; and r>0.9 as very strong. Correlation was considered positive if r>0 and negative if r<0.

## Results

### [18F]-DOPA PET/CT Imaging

**Figure 1:**
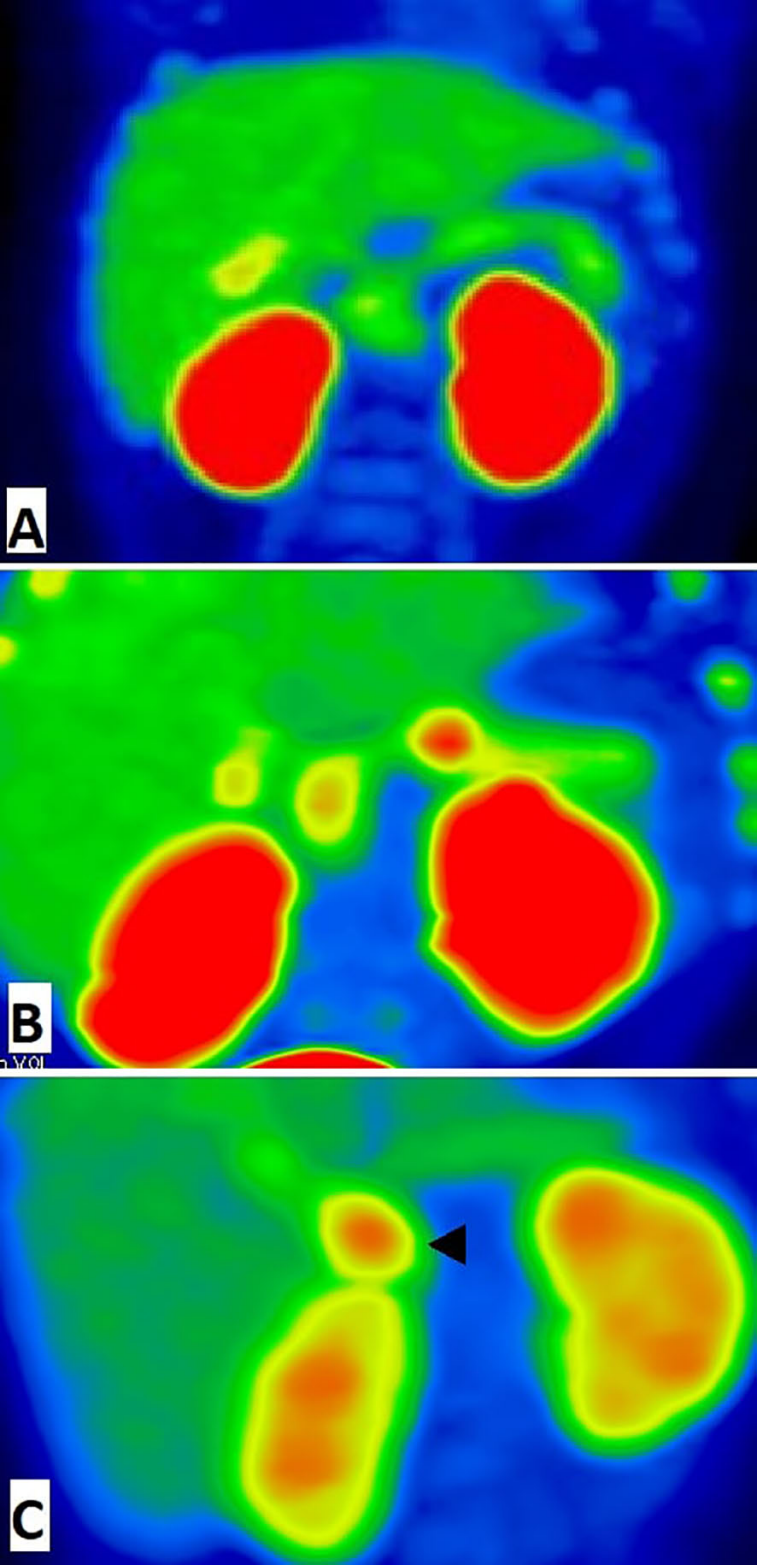
^18^F-fluoro-L-dihydroxyphenylalanine (^18^F-DOPA)-positron emission tomography images (MIP) of pancreas. **(A)** The patient with diffuse congenital hyperinsulinism: homogenic 18F-DOPA uptake in pancreatic tissue. **(B)** The patient with focal form of congenital hyperinsulinism: focal intense ^18^F-DOPA -PET accumulation in the pancreatic body and physiological ^18^F-DOPA uptake in the pancreatic head. **(C)** False positive result of ^18^F-fluoro-L-dihydroxyphenylalanine (18F-DOPA)-positron emission tomography. Histologically proven atypical form imitates focal form of congenital hyperinsulinism with focus of adenomatous hyperplasia in the pancreatic head (arrowhead). In this case, we have found a disagreement between imaging (focal) and pathology (atypical).

Preoperative imaging by [18F]-DOPA PET/CT was consistent with a focal form of СHI in all cases. The indicated foci were confirmed by histology of surgical samples, and in each case the indicated localization of a hyperinsulinemic focus, indicated by PET, corresponded to the actual morphological localization. The most frequent localization of focal adenomatosis was in the pancreatic head (13 patients). Two patients had pathological foci localized in the tail, and 4 patients had a focus in the body of the pancreas (**Figure 1**). A diffuse finding by [18F]-DOPA PET/CT was concordant with the diffuse morphological form of СHI in 9/10 cases. Only one patient with a histologically-proven diffuse form (patient 21 with a heterozygous mutation in the HNF4A gene) had an inconclusive [18F]-DOPA PET/CT result due to high physiological uptake of [18F]-DOPA in the head of pancreas.

The atypical form of СHI appeared to be the most difficult to find by [18F]-DOPA PET/CT. Four cases (patients 31, 32, 33, 35) were histologically confirmed as atypical forms, yet mistakenly interpreted as focal or multifocal forms by PET data. Moreover, one inconclusive PET/CT result (patient 34) was noted in a case of histologically-proven atypical СHI.

Thus, preoperative diagnostics by [18F]-DOPA PET-CT turned out to be highly accurate in focal and diffuse forms of congenital hyperinsulinism, but failed in atypical forms.

### Surgery

All children with CHI-D underwent subtotal pancreaectomy (97-99% of pancreatic tissue removed). In CHI-F, the volume of surgery varied from patient to patient. Those children in whom the focus was located in the head underwent either subtotal head resection (7 patients) or resection of the head and body (i.e., resection of 60-75% of pancreatic tissue) (6 patients). Three out of four patients with pancreatic foci underwent body resection, and one child underwent subtotal resection of 95% of pancreatic tissue. One of the patients with a focus in the tail of the pancreas underwent a tail resection; the other underwent a tail and body resection. Children with CHI-A underwent various operations: resection of the pancreatic head (patient 31 with focal lesions of the head); resection of the head and body (65% of the tissue) (patient 35 with minimal changes in the tissue of the pancreas); resection of the body and tail (patient 33 with multifocal body and tail lesions); or subtotal resection of 95% of the tissue (patient 32 with widespread tissue lesions and patient 34 with minimal changes in the pancreas).

### Light Microscopy

#### Intraoperative Examination of the Pancreas

**Figure 2:**
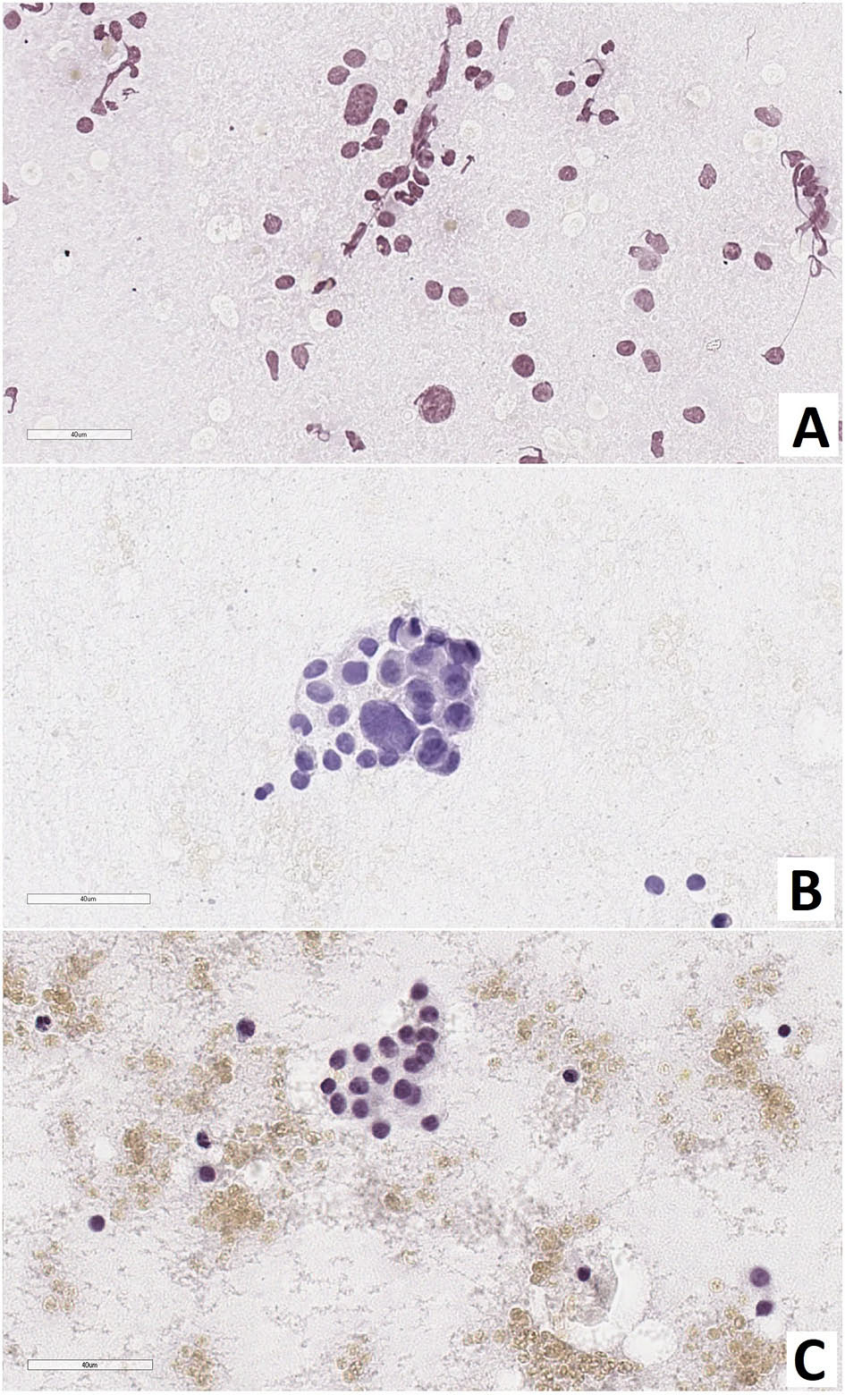
Pancreas smear prints. Large nuclei are 2-3 times larger than neighboring nuclei. **(A)** – focal form of congenital hyperinsulinism, **(B)** – diffuse form of congenital hyperinsulinism, **(C)** – control. Hematoxylin and eosin staining.

Intraoperative examination of pancreatic smears showed the presence of large nuclei (2-3 times larger than neighboring nuclei), on average, in: 14.5 ± 3.5 cells of CHI-F; 8.4 ± 1.1 of CHI-D; and 4.5 ± 0.7 of control group pancreases (from 10 fields of view, 400x, **Figure 2**).

**Figure 3:**
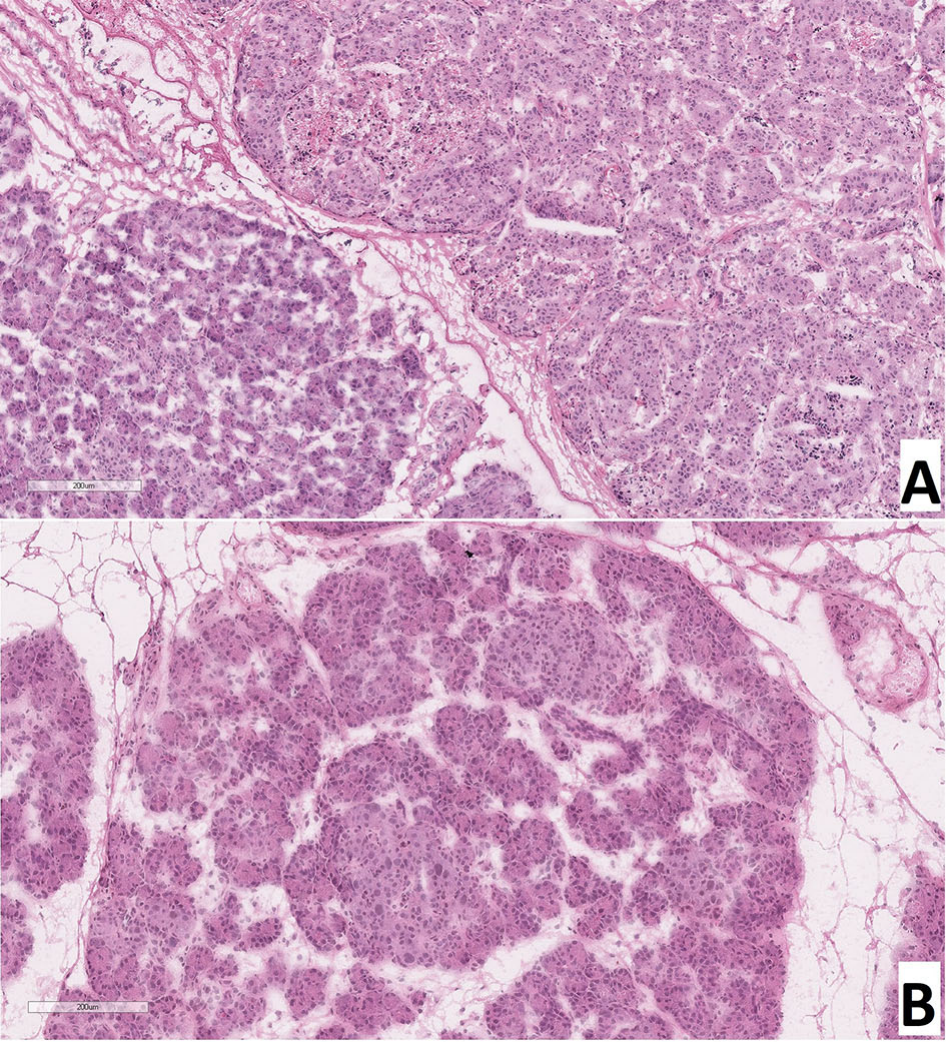
Frozen pancreatic sections. **(A)** – focal form of congenital hyperinsulinism (patient 5), zone of adenomatous hyperplasia with enlarged nuclei of endocrinocytes (right) and normal pancreatic tissue (left). **(B)** – diffuse form of congenital hyperinsulinism (patient 20), enlarged nuclei of endocrinocytes in pancreatic islets. Hematoxylin and eosin staining.

As a result of counting the number of endocrinocytes with hypertrophied nuclei in frozen sections, we found that: with CHI-F, in the zone of adenomatous hyperplasia, there were 5.08 ± 1.36 such cells in the field of view and 0.24 ± 0.21 such cells outside the node; with CHI-D, there were 4.02 ± 1.56 cells; and in control, there were 0.65 ± 0.21. Thus, both in the zone of adenomatous hyperplasia in CHI-F and CHI-D, the number of endocrinocytes with enlarged nuclei was significantly higher both in comparison with the control (p <0.05) and compared with unaffected areas of CHI-F pancreatic tissue (p <0.01, **Figure 3**).

**Figure 4:**
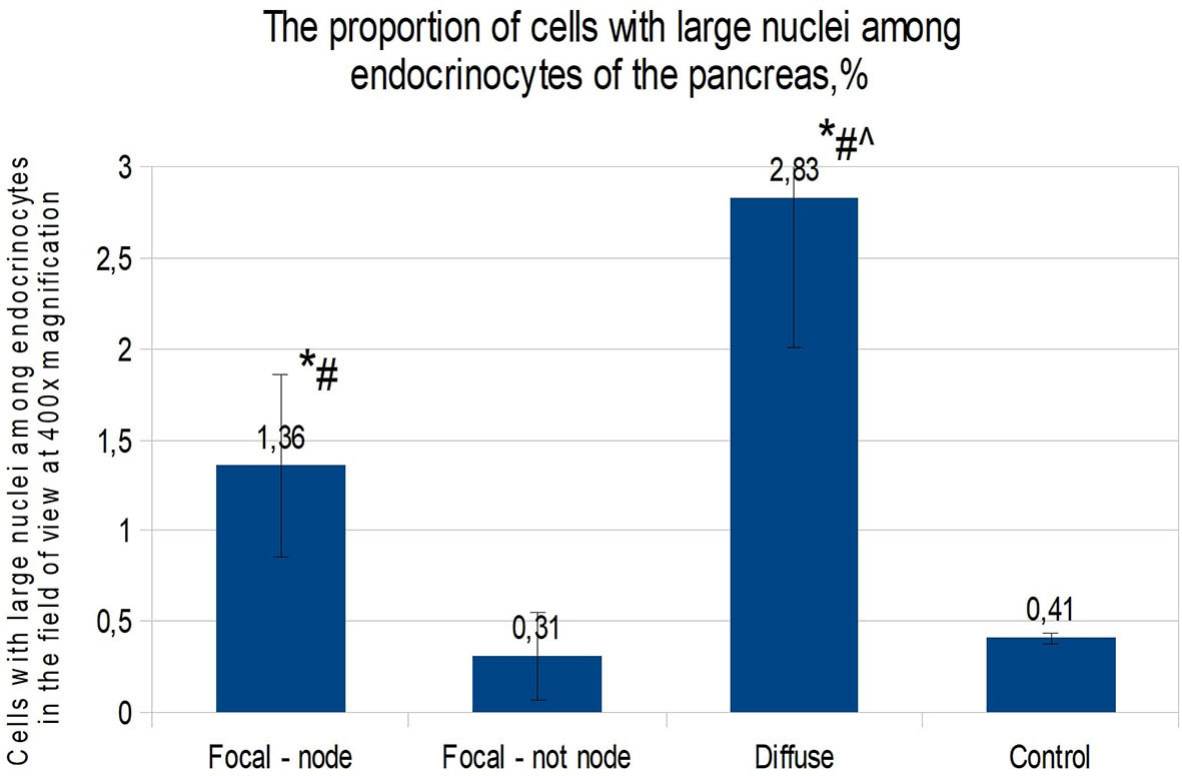
The proportion of cells with enlarged nuclei among endocrinocytes in the field of view (percentage), frozen sections. Data are presented as M ± SD. (*) - the difference in comparison with the control is statistically significant at p <0.05; (#) - the difference in comparison with tissue of the pancreas outside the lesion in the focal form is statistically significant at p <0.01; (^) - the difference compared with the zone of adenomatous hyperplasia is statistically significant at p <0.01. Note: focal node - zone of adenomatous hyperplasia in focal form; focal not node - pancreatic tissue outside the zone of adenomatous hyperplasia; diffuse - diffuse form.

When calculating the proportion of cells with large nuclei (among the total number of endocrinocytes in the field of view), the proportions of cells featuring more than a twofold increase in nuclear size were as follows: in the affected area of the pancreas in CHI-F, 1.36 ± 0.50% of endocrinocytes; outside the affected area in CHI-F, 0.31 ± 0.24%; with CHI-D, 2.83 ± 0.82%; and in the control, 0.41 ± 0.03% (**Figure 4**).

### Histological Examination of Paraffin Sections

#### Control Group

The relative number of endocrinocytes with large nuclei in the pancreas in children was 0.44 ± 0.24%.

### Patients With a Focal Form of Congenital Hyperinsulinism

**Figure 5:**
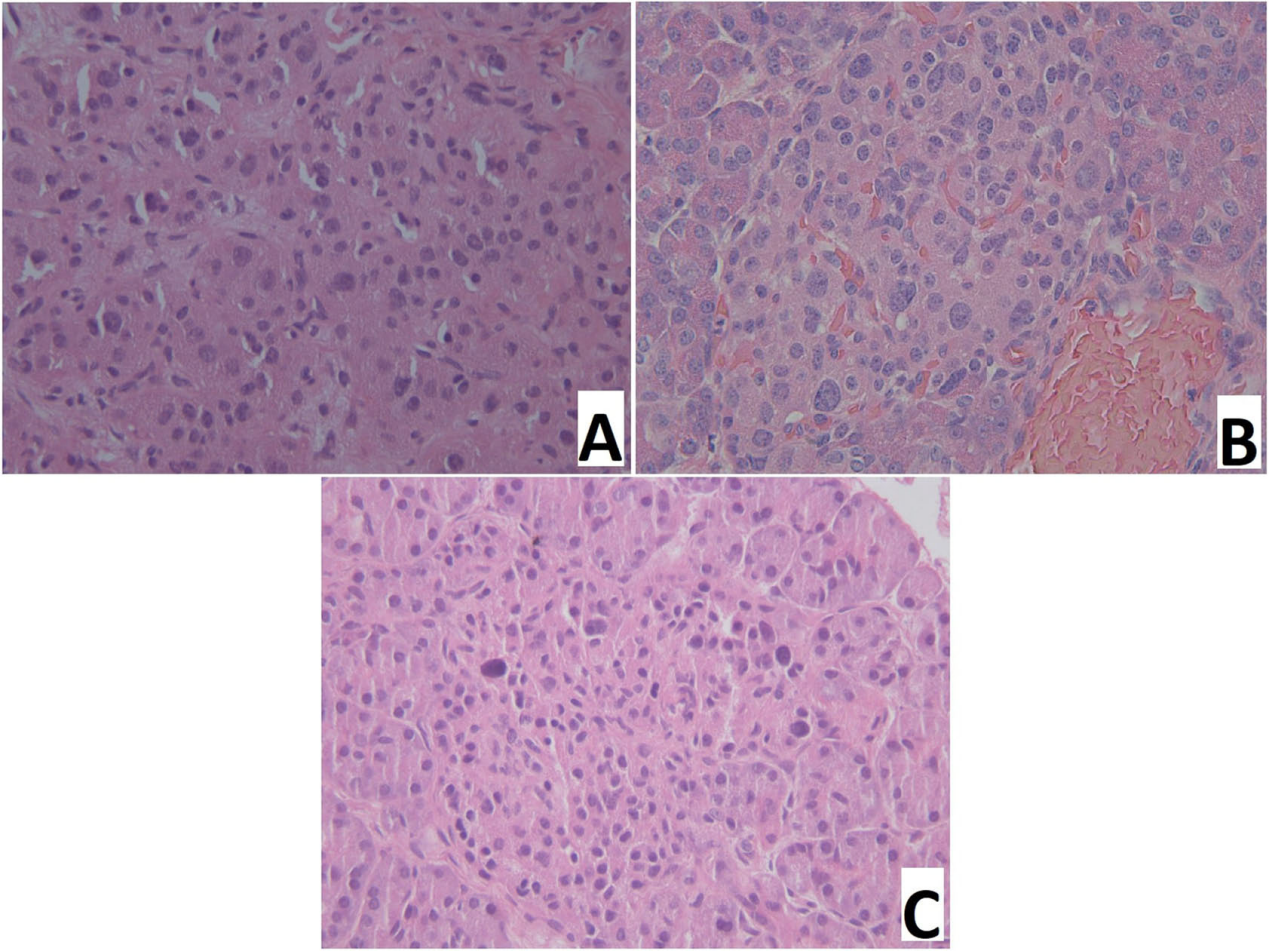
Pancreatic paraffin sections of patients with congenital hyperinsulinism. **(A)** – focal form, a site of adenomatous hyperplasia with hypertrophied endocrinocyte nuclei. **(B)** – diffuse form, an islet of Langerhans with hypertrophied endocrinocyte nuclei. **(C)** – atypical form, islet of Langerhans with hypertrophied nuclei of endocrinocytes. Hematoxylin and eosin staining, x 400.

Children with CHI-F (n = 19) were characterized by the presence of a single node of adenomatous hyperplasia, 0.4-1.1 cm in diameter, in the pancreas, most often localized in the head and somewhat less often in the body and tail. This node consisted primarily of clusters of endocrine cells that formed trabecular or nested structures. In some instances, there were single acini and ducts between the groups of endocrinocytes. Nodes of adenomatous hyperplasia did not have a capsule and were indistinctly delimited from surrounding healthy tissue (**Figure 5A**). In the adenomatous area, endocrinocytes with enlarged nuclei were detected. The relative number of endocrinocytes with large nuclei in the area of adenomatous hyperplasia was 1.82 ± 0.46%. Outside the affected area, they represented 0.58 ± 0.34%. The proportion of endocrinocytes with hypertrophied nuclei in the affected area of the pancreas was statistically significantly higher than that in surrounding healthy tissue or in the control (both with p <0.01). Although the number of endocrine cells with enlarged nuclei outside the node of adenomatous hyperplasia did not generally differ from the control group, endocrinocytes with large nuclei were found somewhat more often in pancreatic regions adjacent to the affected area than in the rest of the endocrine tissue.

**Figure 6:**
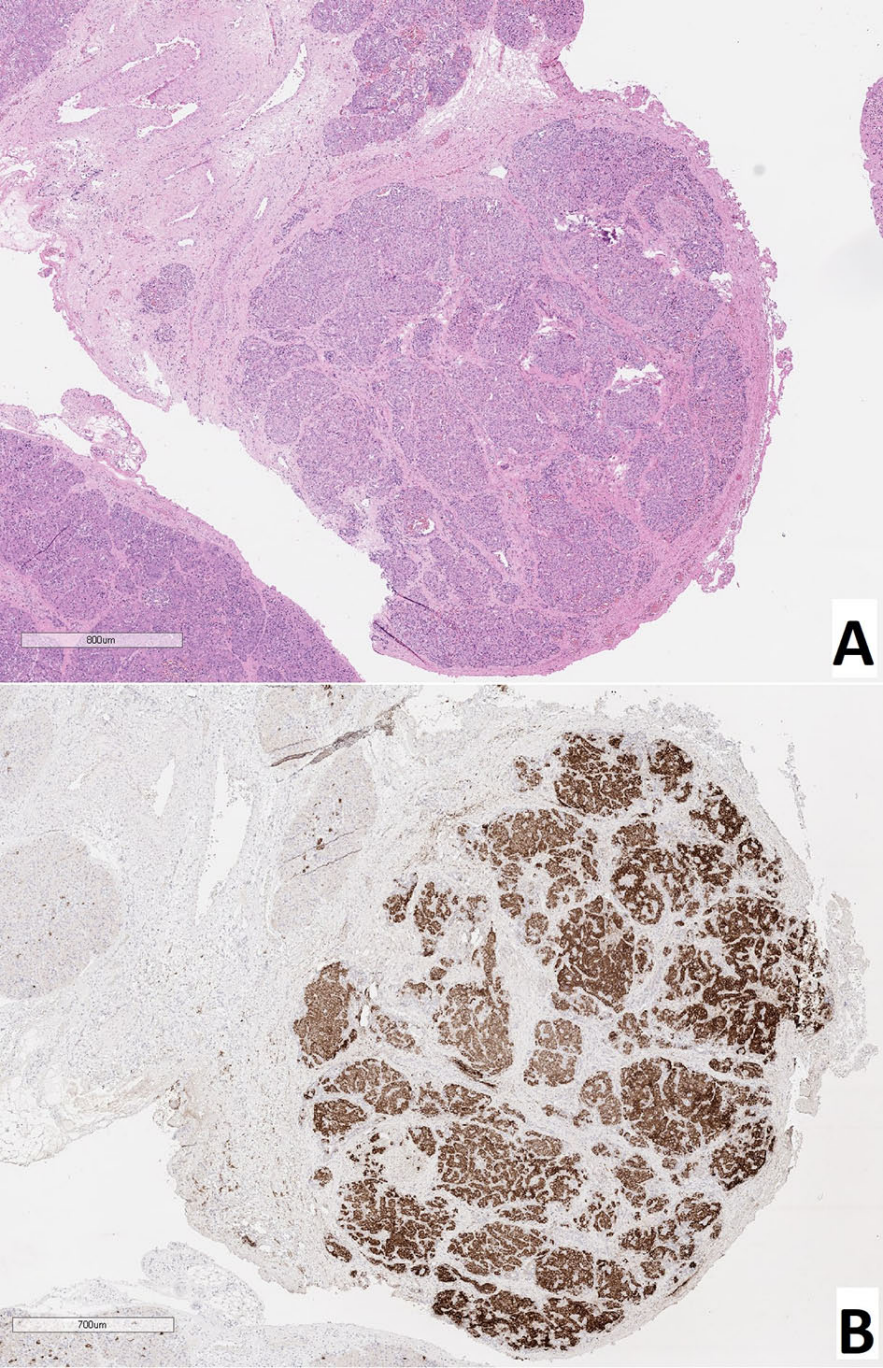
True adenoma in the pancreas of a patient with a focal form of congenital hyperinsulinism. **(A)** – hematoxylin and eosin staining, **(B)** – insulin.

Patient 9 had a multifocal lesion. In the body of the pancreas, 2 nodes of adenomatous hyperplasia were identified. In two patients (4, 11) with a focus in the head, a true adenoma was diagnosed; these were characterized by a homogeneous sinusoidal-trabecular structure and the presence of a thin connective tissue capsule (**Figure 6**). Patient №4 had a KCNJ11 heterozygous mutation, and patient №11 had an ABCC8 heterozygous mutation (**Table 1**).

### Patients With a Diffuse Form of Congenital Hyperinsulinism

In all patients (n = 11), the same type of changes was observed in all areas of pancreatic tissue. In particular, in addition to islets of Langerhans of normal size, large hyperplastic islets were identified. In addition, endocrinocytes with hypertrophied nuclei were found in islets of Langerhans of any size (**Figure 5B**). Their relative amount in the field of view was 4.34 ± 1.66% and was higher than in the control (statistically significantly).

### Patients With an Atypical Form of Congenital Hyperinsulinism

Among children diagnosed with CHI-A (n = 5), histological changes in pancreatic tissues were the most heterogeneous. In patient 31 specifically, numerous hyperplastic islets of Langerhans were observed within the head of the pancreas. These merged with each other in places, but did not form a clear node of adenomatous hyperplasia characteristic of the focal form (**Figure 5C**). Thus, the histological architecture of the lesion was more like CHI-D, and the volume of the lesion was more like CHI-F. In the islets of Langerhans in the affected area, there were many endocrinocytes with hypertrophied nuclei: 5.3 cells in the field of view (3.21% of endocrinocytes in the field). The histological architecture of the pancreas of patient 32 was similar to that of patient 31; numerous enlarged islets of Langerhans merged in places with each other, showing a tendency towards the formation of adenomatous structures. However, in this child, the lesion covered the entire tissue of the pancreas, where the enlarged nuclei of endocrinocytes were detected (2.3 cells in the field of view, or 2.85% of endocrinocytes). The limited area of the pancreatic lesion, and the absence of enlargement of the pancreas in general, made it possible to exclude Beckwith-Widemann syndrome. Patient 32 was born with a body weight of 2260 g, with poor weight gain. She had facial dysmorphism, microcephaly, multiple hyper- and hypopigmented skin lesions, epilepsy, with erythroid and eosinophilic dyspoiesis. Full exome sequencing revealed a homozygous frame shift mutation (c.3535delA: p.Q1178fs (rs1567062835)) in the BLM gene (OMIM №210900). The child was diagnosed with a combination of Bloom’s syndrome and HI. In patient 33, the histological architecture of changes in the pancreas did not differ from that in patient 31. However, the lesion was (2 lesions were identified). In this child, in contrast to patients 31 and 32, relatively few cells with enlarged nuclei were found, even in the affected areas (1.5 cells in the field of view, or 1.37% of endocrinocytes).

In two cases (patients 34 and 35), pancreatic tissue practically did not differ from normal, and the number of endocrinocytes with hypertrophied nuclei was comparable to the control (0.90% and 0.34% of endocrinocytes, respectively). Only in patient 34 was a single microfocus of adenomatous hyperplasia, less than 1 mm in diameter, detected.

**Figure 7:**
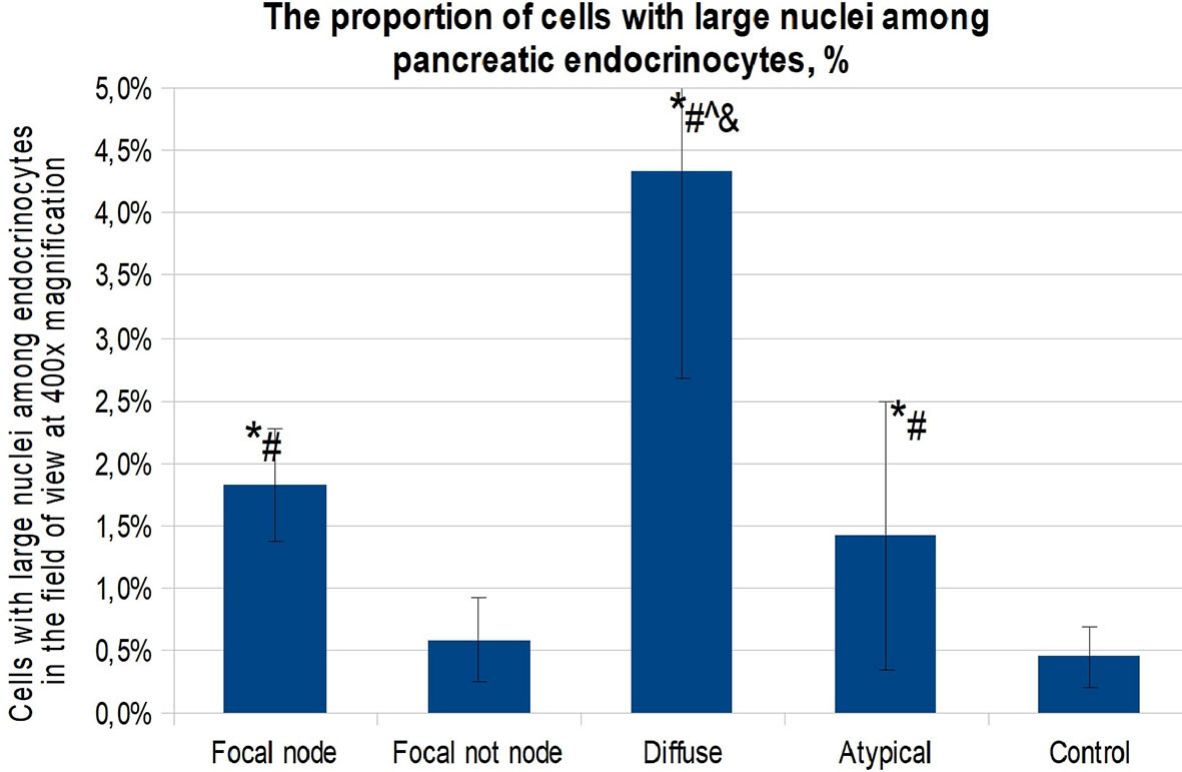
The proportion of cells with enlarged nuclei among endocrinocytes in the field of view (percentage), paraffin sections. Data are presented as M ± SD. (*) - the difference in comparison with the control is statistically significant at p <0.01; (#) - the difference in comparison with the area of unchanged tissue in the focal form is statistically significant at p <0.01; (^) - the difference in comparison with the zone of adenomatous hyperplasia is statistically significant at p <0.01; (&) - the difference compared to the atypical form is statistically significant at p <0.01. Note: focal node - zone of adenomatous hyperplasia in focal form; focal not node - pancreatic tissue outside the zone of adenomatous hyperplasia; diffuse - diffuse form; atypical - atypical form.

Thus, the CHI-A patient group was extremely heterogeneous, and each such case required a separate morphological assessment. The proportion of endocrinocytes with large nuclei in patients with CHI-A was 1.42 ± 1.08%. This was statistically significantly (p <0.01) higher compared to the control and pancreatic tissue outside the adenomatous hyperplasia node in CHI-F (**Figure 7**).

Thus, endocrinocytes with enlarged nuclei were observed in all morphological forms of CHI. It was found that in the islets of Langerhans in CHI-D, endocrinocytes with enlarged nuclei are found statistically significantly (p <0.01) more often than in the control, CHI-A, or CHI-F (inside or outside the zone of adenomatous hyperplasia). In addition to the above, it was found that there is a statistically significant (p <0.05) moderate inverse correlation between the age of patients and the proportion of endocrine cells with hypertrophied nuclei (rs = -0.436). That is, the older the child was at the time of surgery, the lower the percentage of endocrinocytes containing large nuclei.

### Immunohistochemical Study

#### Patients From the Control Group

**Figure 8:**
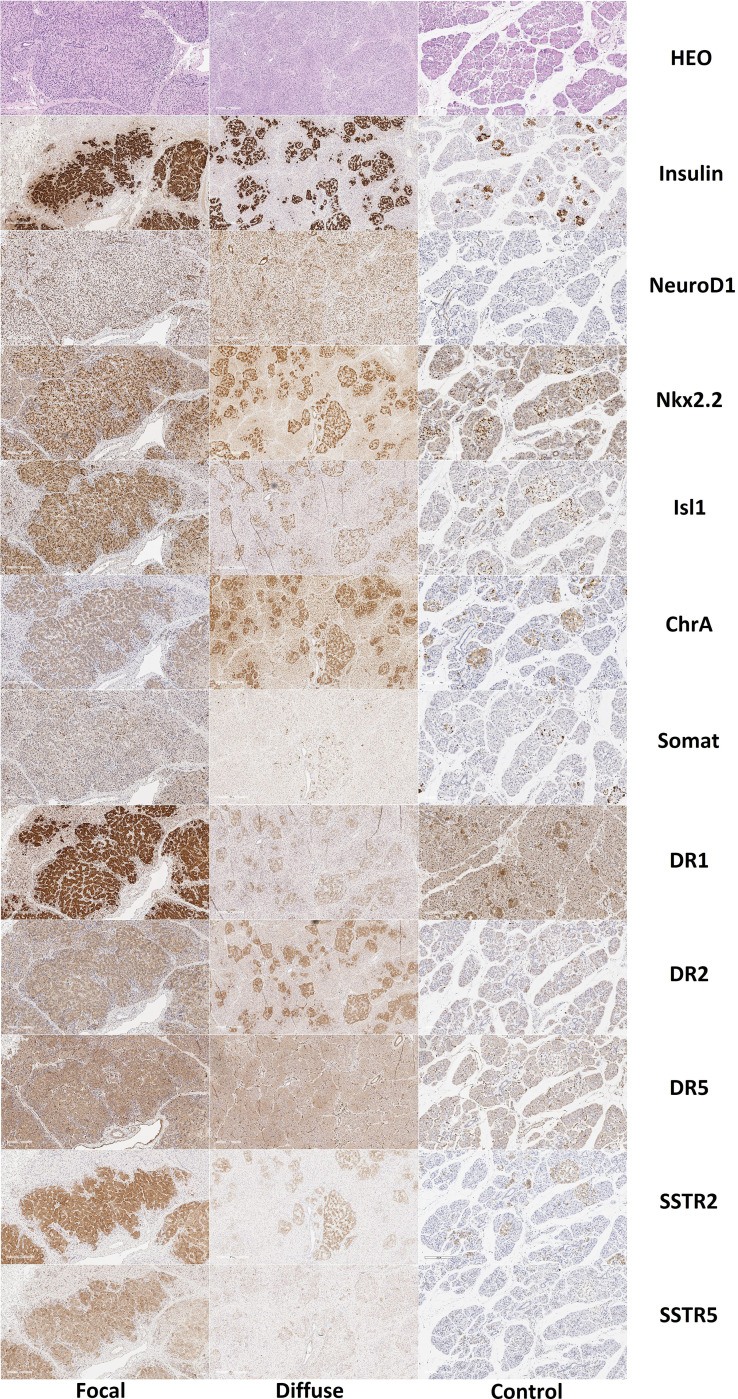
Histological and immunohistochemical examination of pancreatic tissue in patients with congenital hyperinsulinism and in controls. Hematoxylin and eosin staining, insulin, NeuroD1 and Nkx2.2, Isl1, chromogranin A, somatostatin and DR1, DR2, DR5, SSTR2 and SSTR5; x50. Note: HEO – hematoxylin and eosin, Somat – somatostatin, ChrA – chromogranin A.

Expression of chromogranin A, insulin, Isl1, and Nkx2.2 was observed in endocrinocytes of the islets of Lagerhans and single cells outside them (**Figure 8**). SST was expressed in cells at the periphery of endocrine islets and in single cells outside of them. Immunohistochemical staining for NeuroD1 was positive in the cells of the islets of Langerhans only in 1 child and only in 7% of endocrinocytes. Type 1 dopamine receptors (DR1) were found almost exclusively in the endocrine pancreas. In some cases, antibodies to DR1 stained the cytoplasm of exocrinocytes or excretory ducts. DR2 was expressed predominantly in the endocrine pancreas. In all cases, the immunohistochemical staining was cytoplasmic. Moreover, in some cases, the cytoplasm of endocrinocytes was evenly stained and, in some cases, numerous brown granules were detected in it. In some patients, such granules were found not only in the endocrine, but also in the exocrine part of the pancreas. DR5 was only detected in the cytoplasm of blood vessel endothelium; in some patients, there was detection in the cytoplasm of excretory ducts. Endocrine and exocrine cells were not stained with anti-DR5 antibodies. SSTR2 and SSTR5 were detected predominantly in the endocrine pancreas in all children.

### Patients With the Focal Form of Congenital Hyperinsulinism

**Figure 9:**
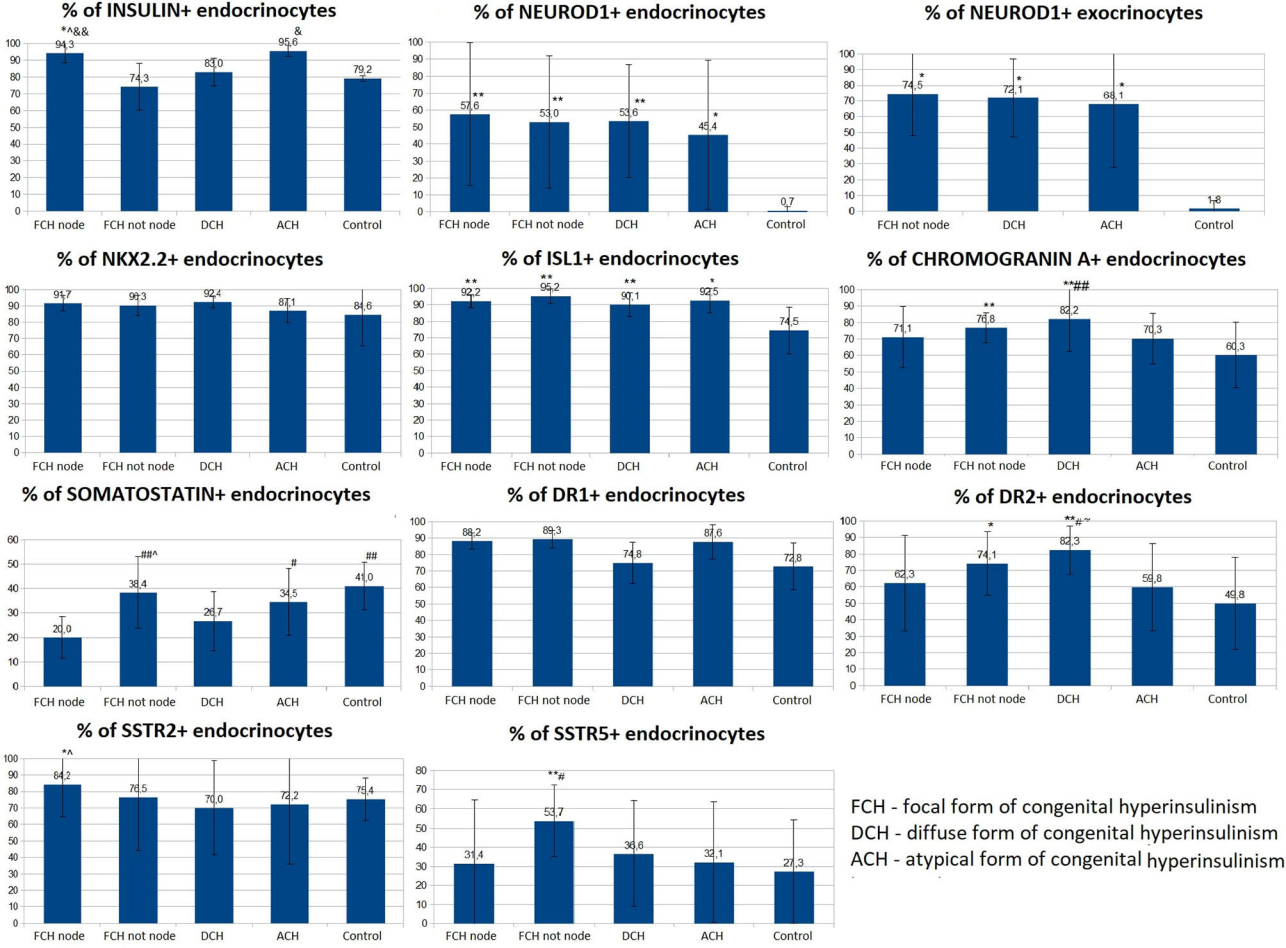
The proportion of endocrinocytes expressing various markers in pancreatic tissue in congenital hyperinsulinism and in control. Data are presented as M ± SD. (*) and (**) - the difference in comparison with the control is statistically significant at p < 0.05 and p < 0.01 respectively; (#) and (##) - the difference in comparison with the zone of adenomatous hyperplasia is statistically significant at p < 0.05 and p < 0.01 respectively; (&) and (&&) - the difference in comparison with the area of unchanged tissue in the focal form (CHI-F outside the node) is statistically significant at p < 0.05 and p < 0.01 respectively; (^) - the difference compared to the diffuse form is significant at p < 0.05.; (~) - the difference compared to the atypical form is significant at p <0.05.

Like the control group, expression of chromogranin A, insulin, Isl1, Nkx2.2, and SST was observed in the endocrinocytes of the islets of Lagerhans and single cells outside them. The proportion of endocrinocytes expressing chromogranin A was 71.10 ± 18.41% in the node of adenomatous hyperplasia and 76.83 ± 9.22% outside of it (**Figure 9**). The proportion of endocrinocytes expressing insulin was 94.26 ± 5.83% in the affected area and 74.3 ± 16.74% outside of it. As seen in **Figure 8**, the other values were (affected, outside): 92.17 ± 4.07% and 95.22 ± 4.28% for Isl1; 91.70 ± 4.65% and 90.25 ± 6.17% for Nkx2.2; 20.02 ± 8.63% and 38.36 ± 14.64% for SST. It is important to note that in the unchanged area of the pancreas, the proportion of endocrinocytes stained with antibodies to chromogranin A turned out to be statistically significantly (p <0.01) higher than in the control group, and the proportion of SST-positive endocrine cells in the area of adenomatous hyperplasia was statistically significantly lower than outside and in control (p <0.01).

**Figure 10:**
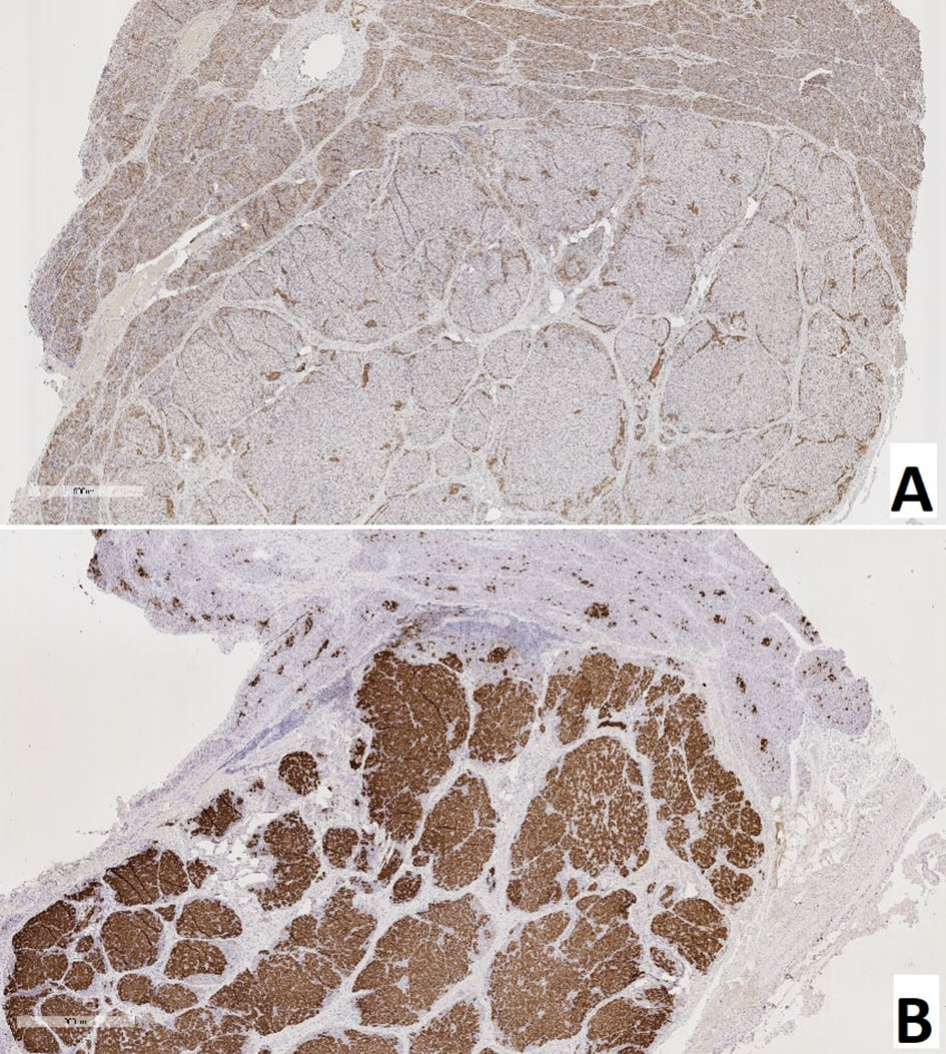
Pancreas of a patient with a focal form of congenital hyperinsulinism. **(A)** – NeuroD1, **(B)** – insulin; x50.

The proportion of NeuroD1-positive endocrinocytes in the affected area was, on average, 57.64 ± 41.96%. In 7 patients however, the range of values was 2-20% (on average 10.03 ± 7.13%). In the remaining 10 patients, 66.5 - 100.0% of cells expressed NeuroD1 (on average 90.95 ± 10.13%). In 3 of the 7 patients mentioned above (11, 14, 16), expression of NeuroD1 was detected only along the contour of the node of adenomatous hyperplasia or was not detected at all (**Figure 10**). In general, in patients with CHI-F, expression of NeuroD1 in the endocrine tissue of the zone of adenomatous hyperplasia was statistically significantly (p <0.01) higher than in the control.

**Figure 11:**
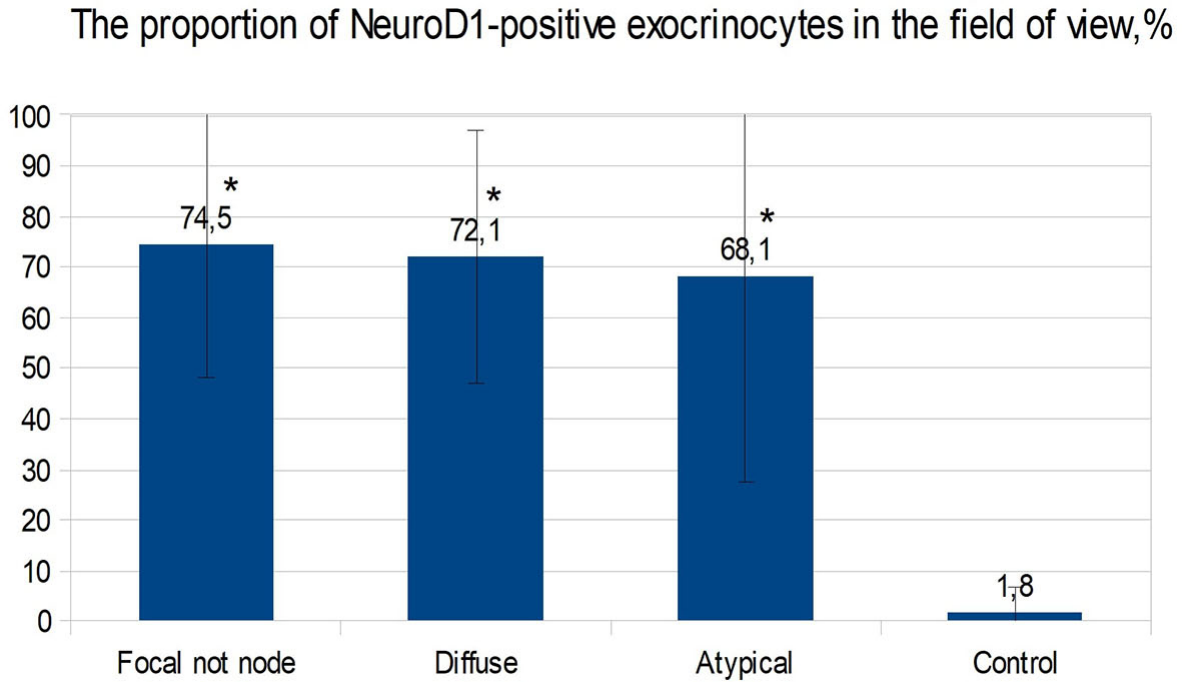
The proportion of exocrinocytes expressing NeuroD1 in the field of view in various forms of congenital hyperinsulinism. Data are presented as M ± SD. (*) - the difference compared to the control is statistically significant at p <0.01.

In the islets of Langerhans outside the affected area, it was found that expression of NeuroD1 is observed in 53.00 ± 39.04% of endocrinocytes, which is statistically significantly higher compared to the control (p <0.01). In addition, a highly statistically significant (p <0.05) positive correlation (rs = 0.703) was found between the number of NeuroD1-positive endocrinocytes in the affected area of the pancreas and unaffected areas. Among endocrinocytes with enlarged nuclei, the proportion of cells expressing NeuroD1 practically did not differ from the proportion of all NeuroD1-positive endocrinocytes in the field of view: 50.51 ± 38.22% *versus* 57.64 ± 41.96%. Evaluation of the expression of this transcription factor in the exocrine pancreas revealed a statistically significant (p <0.01) increase in the proportion of NeuroD1-positive acinar cells compared with the control. Outside the zone of adenomatous hyperplasia, NeuroD1 was expressed in 74.52 ± 26.44% of exocrinocytes (**Figure 11**).

Dopamine receptors were expressed in the same places as in the control group, only more intensely. The proportion of DR1-positive endocrine cells was 88.23 ± 4.88% in the affected area and 89.33 ± 5.13% outside; it did not differ statistically significantly from the control. The relative proportion of DR2-positive endocrine cells was 62.27 ± 29.08% in the affected area and 74.11 ± 19.24% outside it. In addition, outside the adenomatous hyperplasia, the proportion of endocrinocytes stained with antibodies to DR2 was statistically significantly (p <0.05) higher than in the control. In some patients, granular expression of this antigen was found not only in the endocrine, but also in the exocrine part of the pancreas. DR5, as in the control, was not expressed on endocrinocytes.

The proportion of endocrinocytes expressing SSTR2 was 84.24 ± 19.75% in the affected area and 76.47 ± 32.06% outside it; in the node of adenomatous hyperplasia, it was statistically significantly higher (p <0.05) than in control islets of Langerhans. Nevertheless, in patient 7, the proportion of SSTR2-positive cells was significantly lower than in the control: 23.7% in the area of adenomatous hyperplasia and 25.8% outside it (some islets of Langerhans were stained with antibodies to SSTR2 almost completely, while others did not stain at all).

In another patient (8), SSTR2 was detected only in 3.2% of endocrine cells outside the affected area. The intensity of immunohistochemical staining with SSTR5 was much weaker than to SSTR2. The proportion of endocrinocytes expressing SSTR5 was 31.39 ± 33.07% in the affected area and 53.73 ± 18.65% outside it. Moreover, the relative numbers of SSTR5-positive endocrine cells in the area of adenomatous hyperplasia and in the control were statistically significantly lower than in unaffected CHI-F pancreatic areas (p <0.01 and p <0.05, respectively). At the same time, there was no correlation between the number of SSTR5-positive endocrinocytes in the node of adenomatous hyperplasia and outside it. SSTR5 was not expressed in 3 patients (22, 25, 28).

### Patients With the Diffuse Form of Congenital Hyperinsulinism

For all immunohistochemical markers, the localization and degree of expression intensity did not differ from those in the CHI-F patient group. Expression of NeuroD1 also prevailed in the exocrine pancreas, in contrast to the control group. The proportion of endocrinocytes expressing chromogranin A was 82.21 ± 19.85%. At the same time, the proportion of chromogranin A-positive endocrine cells in patients with CHI-D was statistically significantly (p <0.01) higher than in the control or in the node of adenomatous hyperplasia in CHI-F. The relative proportions of marker-expressing endocrinocytes were: insulin 82.98 ± 8.31%; Isl1 90.09 ± 7.13%; Nkx2.2 92.42 ± 3.73%; and SST 26.69 ± 12.17%. In addition, the proportion of endocrinocytes stained by SST antibodies was lower than in unaffected pancreatic tissue in CHI-F (significant at p <0.05); the proportion was also lower than in controls (difference not reaching statistical significance).

The proportion of endocrinocytes expressing NeuroD1 averaged 53.62 ± 33.29%, which is statistically significantly (p <0.01) higher than in the control. However, in some patients (especially 26, 29), pronounced mosaicism was observed in staining for NeuroD1: some islets of Langerhans were almost completely NeuroD1-positive, and some were negative. Among endocrinocytes with enlarged nuclei, the proportion of cells expressing NeuroD1 practically did not differ from the proportion of all NeuroD1-positive endocrinocytes in the visual field, as in other forms of CHI. NeuroD1 was expressed in 72.07 ± 24.86% of exocrinocytes. The relative proportion of endocrinocytes with DR1 expression was 74.83 ± 12.46%; the proportion expressing DR2 was 82.33 ± 14.42%. In patients with CHI-D, the proportion of DR2-positive endocrine cells was statistically significantly higher than in controls (p <0.01), CHI-F (in the node of adenomatous hyperplasia or outside it) (p <0.05), or CHI-A (p <0.05). The proportion of endocrinocytes expressing SSTR2 was 69.99 ± 28.59%; the proportion expressing SSTR5 was 36.55 ± 27.73%. In one case (patient 30), SSTR2 and SSTR5 were not expressed. In 8 cases, SSTR5 was not expressed (patients 5, 7, 8, 11, 13, 14, 16, 17).

### Patients With Atypical Form Congenital Hyperinsulinism

The proportions of endocrinocytes expressing markers were chromogranin A (70.26 ± 15.38%), Isl1 (92.52 ± 7.39%), and Nkx2.2 (87.8 ± 7.46%). The largest proportion of insulin-containing endocrinocytes was found in CHI-A (95.55 ± 3.32%). In these patients, 98.48 ± 2.63% of endocrinocytes with hypertrophied nuclei expressed Isl1, which was significantly higher compared to other forms of CHI (p <0.05).

Differences in Nkx2.2 expression by endocrinocytes with hypertrophied nuclei, compared to all endocrinocytes, were also statistically significant (p <0.05): 98.25 ± 3.04% *versus* 87.08 ± 7.46%. In CHI-A, SST expression (34.54 ± 13.65%) did not significantly differ from that in the control or pancreatic tissue outside the adenomatous hyperplasia node; it was significantly higher than with CHI-D (p <0.05) or CHI-F (in the focus of adenomatous hyperplasia) (p <0.05). SST was not detected in endocrinocytes with hypertrophied nuclei, in contrast to CHI-D and CHI-F (0.41 ± 1.37% and 4.04 ± 4.63% of cells, respectively).

In children with CHI-A in the endocrine pancreas, average NeuroD1 expression was significantly higher (p <0.05) than in the control group (45.4 ± 44.2% and 0.70 ± 2.21% respectively). However, the data obtained in different patients with this form were extremely heterogeneous. In particular, two children (patients 34, 35) had no NeuroD1-positive endocrinocytes at all, while in the remaining patients the proportions of endocrine cells stained for NeuroD1 were 83%, 52%, and 92%. It is interesting to note that in patients 34 and 35, histological examination of pancreatic tissue showed only minimal changes. NeuroD1 was expressed in 68.05 ± 40.46% of exocrinocytes.

The relative number of endocrinocytes with DR1 expression was 87.63 ± 10.43%; those expressing DR2 were 59.77 ± 26.53%. Interestingly, in patient 34, the relative number of DR2-expressing endocrinocytes in the microadenoma was much lower than in the islets of Langerhans outside of it (42.7% and 92.1%, respectively). The proportion of endocrinocytes expressing SSTR2 was 72.16 ± 36.22%; the proportion expressing SSTR5 was 32.07 ± 31.50%. In one child (patient 35), SSTR2 was expressed in only 9.2% of endocrinocytes, and SSTR5 was not expressed at all. Patient 34 also did not express SSTR5.

Thus, NeuroD1 has been shown to be the best overall CHI marker. At low magnification, expression patterns of insulin, Isl1, Nkx2.2, and chromogranin A can be used to differentiate disease forms. DR2 can be used for quantitative differential diagnosis of forms on a small volume of material (counting the relative number of cells with expression in the field of view). The number of cells expressing SST is clearly reduced in CHI-D and CHI-F. CHI-A is distinguished by the largest relative number of insulin-expressing endocrinocytes in the field of view, as well as by the presence of a larger number of cells with large nuclei expressing the transcription factors Isl1 and Nkx2.2. SSTR2 is expressed in CHI in the overwhelming majority of cases (97%); SSTR5 is expressed 1.5 times less frequently in cases.

### Fluorescence Microscopy

**Figure 12:**
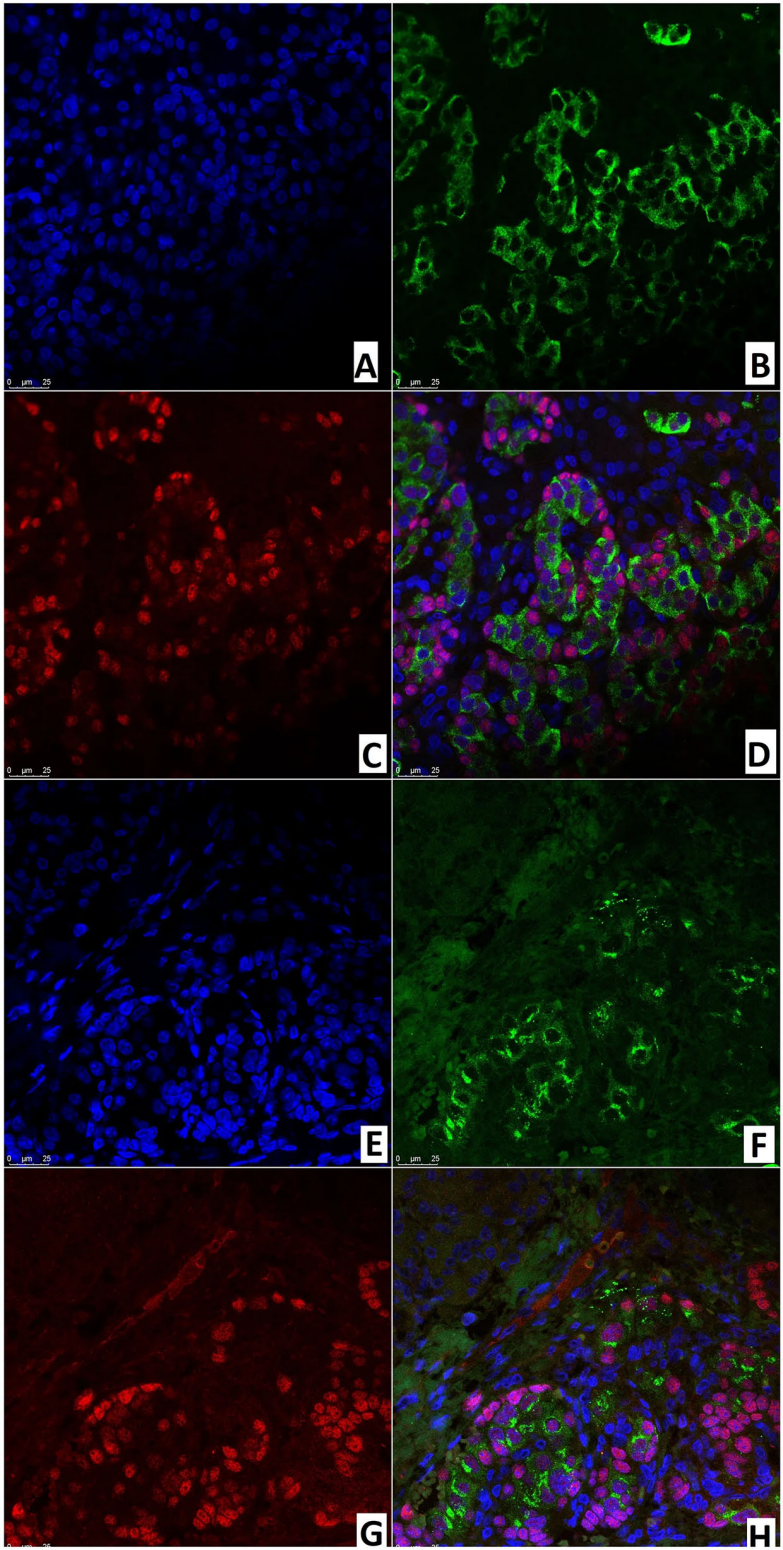
Confocal laser scanning microscopy of the pancreas in congenital hyperinsulinism. **(A–D)** focal form, **(E, F)** diffuse form. **(A, F)** blue fluorescence of cell nuclei (DAPI). **(B, F)** green fluorescence of insulin in cytoplasm. **(C, G)** red fluorescence of Isl1 in nuclei. **(D, H)** Isl1 (rose fluorescence) of the nuclei and insulin (red fluorescence) in the same cells; x400.

Like the immunohistochemical analysis, fluorescence microscopy revealed insulin in the cytoplasm and the transcription factor Isl1 in the nuclei of endocrinocytes. Same-cell insulin/Isl1 colocalization (**Figure 12**) was analyzed in: the node of adenomatous hyperplasia in a patient with CHI-F (70.8 ± 5.5%); and in the islets of Langerhans of a child with CHI-D (82.0 ± 3.6%). In addition, in unaffected islets of Langerhans in CHI-F, insulin-positive cells were located mainly closer to the center of the islet; Isl1-positive cells, on the contrary, were closer to its periphery. Yet with CHI-D, this trend was not observed. However, in a patient with CHI-F, a significant fraction of endocrine cells (19.5%) in the adenomatous hyperplasia node stained only insulin. Outside of it, a significant fraction (44.2%) of endocrinocytes was, on the contrary, only Isl1-positive.

**Figure 13:**
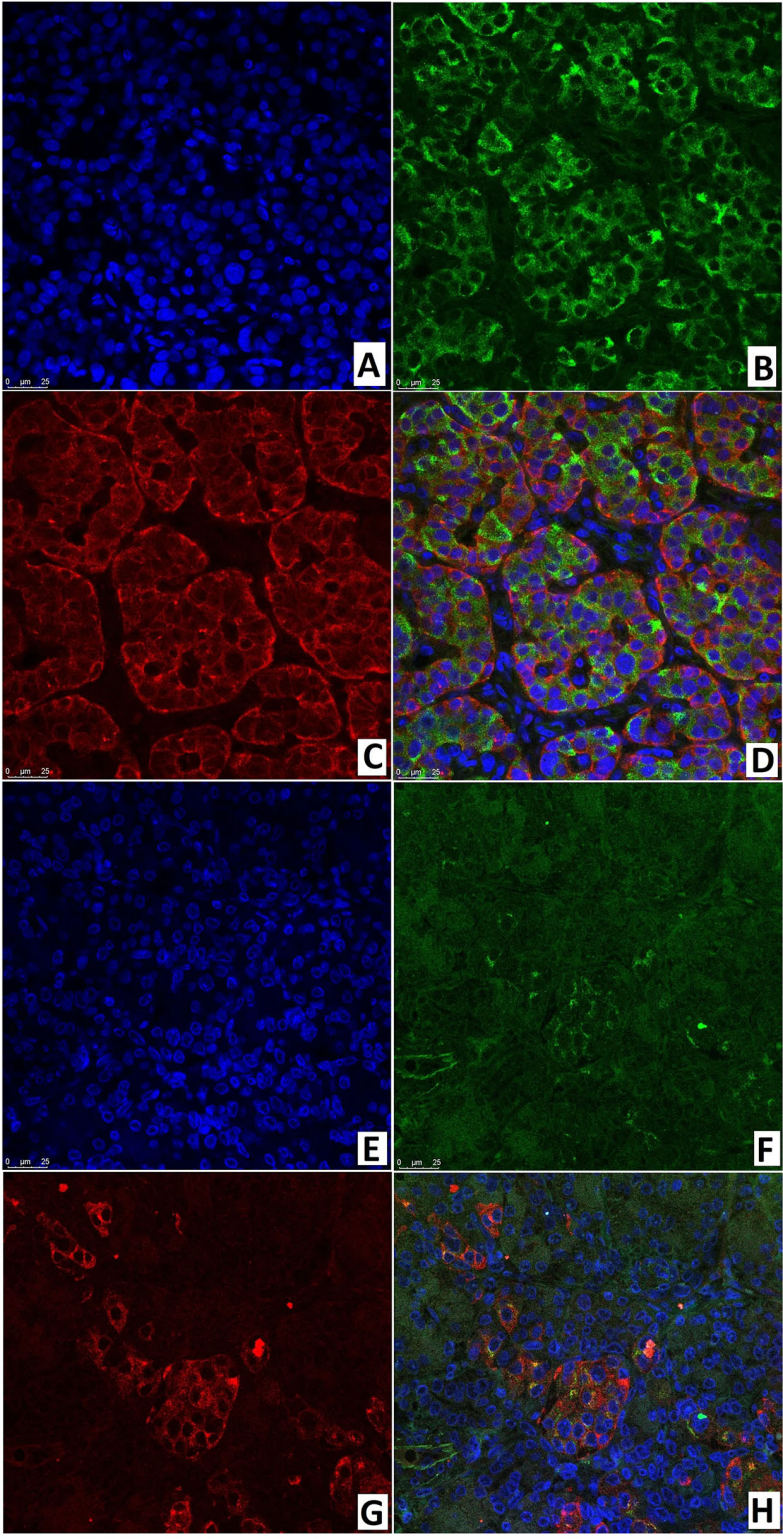
Confocal laser scanning microscopy of the pancreas in congenital hyperinsulinism. **(A–D)** focal form, **(E–H)** diffuse form. **(A, E)**: blue fluorescence of cell nuclei (DAPI). **(B, F)**: green fluorescence of insulin in cytoplasm. **(C, G)**: red fluorescence of SSTR2. **(D, H)**: coexpression of insulin and SSTR2 observed as orange fluorescence; x400.

In the node of adenomatous hyperplasia in a patient with CHI-F, the proportion of insulin/SSTR2 coexpressing cells was 55.6 ± 5.2%. In the islets of Langerhans of a child with CHI-D, it was 62.9 ± 10.8%; in pancreatic tissue outside the zone of adenomatous hyperplasia, it was only 25.0 ± 3.5% (**Figure 13**). The remaining endocrinocytes in the adenomatous hyperplasia node and in CHI-D expressed only insulin twice more often than SSTR2 alone; in a child with CHI-F in the adenomatous node, these were 28.6 ± 5.4% and 15.9 ± 4.7%, respectively. In a CHI-D patient, the proportion of insulin+/SSTR2- cells was 24.8 ± 4.5%, while insulin-/SSTR2+ was only 12.4 ± 3.9%. At the same time, in the unaffected tissue of the pancreas of the patient with CHI-F, the majority of endocrinocytes (68.8 ± 7.6%) were insulin-/SSTR2+; only a few (6.3 ± 4.6%) were insulin+/SSTR2-.

**Figure 14:**
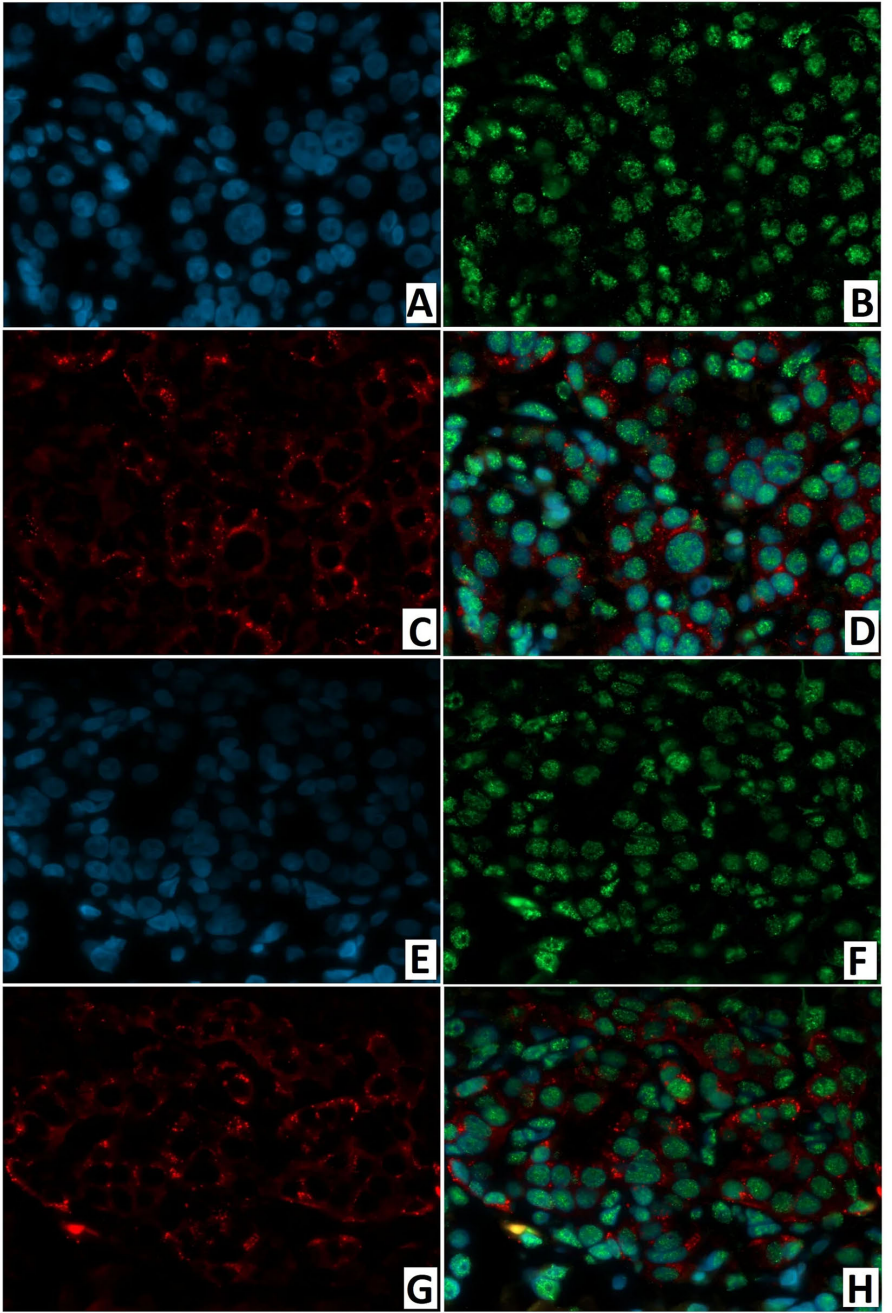
Confocal laser scanning microscopy of the pancreas in congenital hyperinsulinism. **(A–D)** focal form, **(E, F)** diffuse form. **(A, F)** blue fluorescence of cell nuclei (DAPI). **(B, F)** green fluorescence of NeuroD1 in nuclei. **(C, G)** red fluorescence of ChrA in cytoplasm. **(D, H)** greenish-blue/turquoise fluorescence of NeuroD1 and red fluorescence of ChrA in the same cells; x400.

ChrA/NeuroD1 colocalization was detected in 50.0 ± 9.3% of cells in the node of adenomatous hyperplasia in CHI-F (**Figures 14A–D**) and in 61.1 ± 10.6% of cells of the islets of Langerhans in CHI-D (**Figures 14E–H**). Fluorescence microscopy confirmed that NeuroD1 is present not only in endocrine but also exocrine cells.

**Figure 15:**
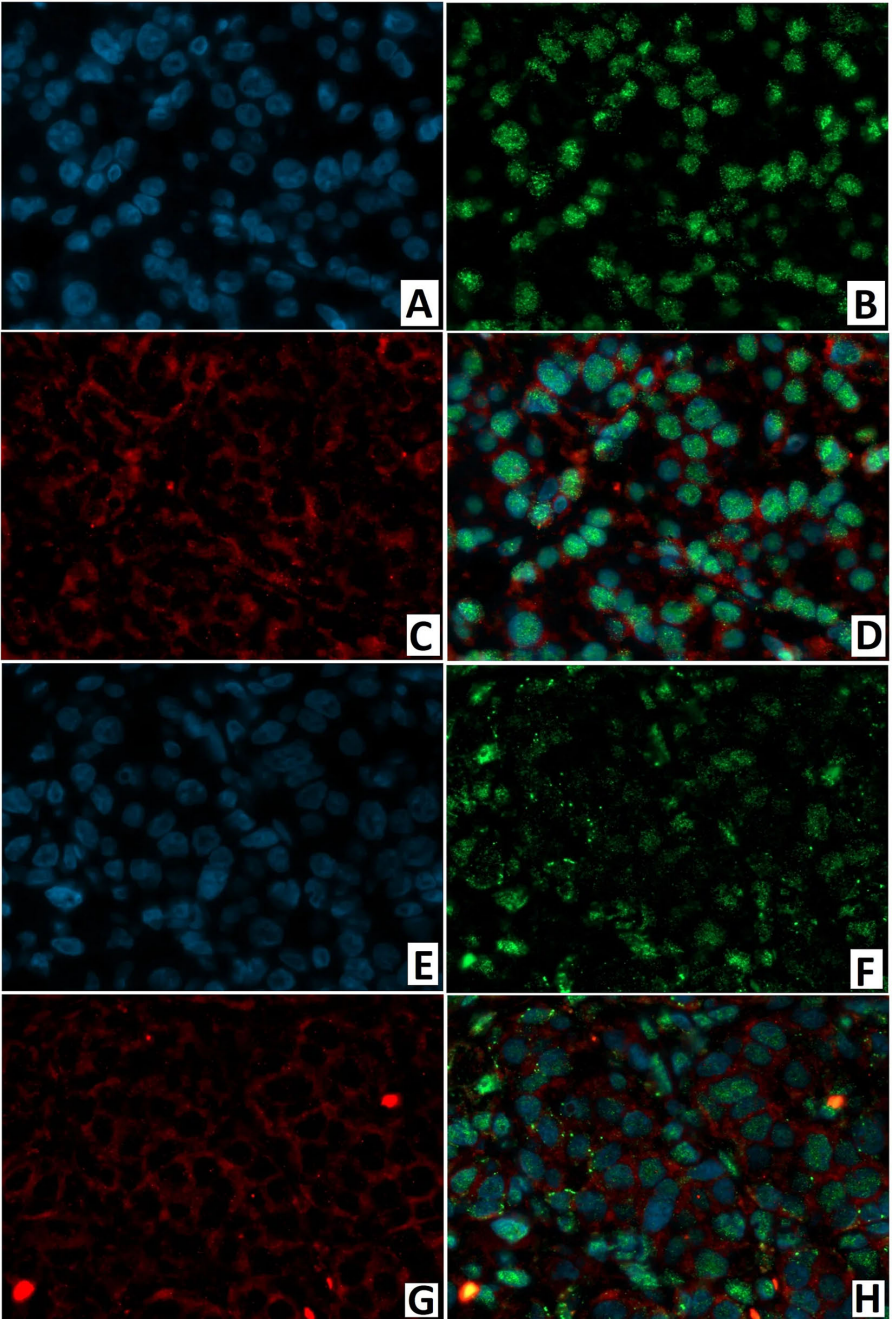
Confocal laser scanning microscopy of the pancreas in congenital hyperinsulinism. **(A–D)** focal form, **(E, F)** diffuse form. **(A, F)** blue fluorescence of cell nuclei (DAPI). **(B, F)** green fluorescence of NeuroD1 in nuclei. **(C, G)** red fluorescence of DR2 in cytoplasm. **(D, H)** greenish-blue/turquoise fluorescence of NeuroD1 and red fluorescence of DR2 in the same cells; x400.

As with immunohistochemical analysis, fluorescence microscopy revealed: DR2 in the cytoplasm of endocrinocytes; and the transcription factor NeuroD1 in the nuclei of endocrine and exocrine cells (**Figure 15**). In a CHI-F patient, in the node of adenomatous hyperplasia, NeuroD1/DR2 colocalization was observed in 61.8 ± 12.4% of cells. In CHI-D, 34.7 ± 6.9% of cells showed the colocalization. In both forms, few endocrine cells expressed only DR2 (from 2.4% to 4.1%).

## Discussion of the Research Results

As a result of morphological examination, 3 forms of CHI were identified: diffuse, adenomatous, and atypical. In 2 cases of CHI-F, true adenomas were diagnosed, which were characterized by a homogeneous sinusoidal-trabecular structure and the presence of a thin connective tissue capsule located among adenomatous structures. The presence of a true adenoma in CHI, as well as denial of this form by some authors (29), has been described by others (30).

In our opinion, it is extremely significant that cells with large, hyperchromic nuclei were observed not only in CHI-D, but also in CHI-A and CHI-F (both inside and outside actual adenomatous structures). This complicates urgent histological analysis for differential diagnosis of CHI forms. Studies by Han et al. (28) also showed that nucleomegaly occurs not only in CHI-D in insulin islets, but also in normal areas of the pancreas in CHI-F patients. The frequency of nucleomegaly was the highest in CHI-D. At the same time, the authors demonstrated that nucleomegaly is not associated with cell proliferation. The presence of large nuclei outside the foci of adenomatous hyperplasia was also noted by Suchi et al. (31). In our study, cells with large nuclei expressed chromogranin A, Isl1, Nkx2.2, NeuroD1, and SST, not just insulin. Moreover, CHI-A was different, with statistically significance, in the highest relative number of cells with nucleomegaly expressing Isl1, Nkx2.2, or insulin in the field of view. This, in our opinion, indicates that nuclear enlargement in CHI is due to the readiness of cells to secrete more insulin (and in some cells SST as well) under the influence of these transcription factors.

Our analysis of Isl1 and Nkx2.2 expression, in different parts of the pancreas from different forms of CHI or normal tissues, confirmed that these transcription factors are synergistic. It is known that Isl1, like NKX2.2, regulates the formation of all hormone-producing cells (32). The latter transcription factor is found in α-, β-, and δ-cells of the human pancreas already at 8–21 weeks of gestation, but it is crucial for maintaining β-cell identity (33). Churchill et al. (34) demonstrated that Nkx2.2 functions as an integral component of a modular regulatory program to correctly specify the fates of pancreatic islet cells.

Nevertheless, it seems to us that among the transcription factors, NeuroD1 is the clearest marker for CHI diagnosis in general. It was found that, when comparing expression levels (Isl1, Nkx2.2, NeuroD1), the greatest difference in islets of Langerhans (CHI compared with normal/control pancreas) was observed precisely for NeuroD1.

Contrary to literature data indicating that NeuroD1, an insulin transactivator, is necessary for the maturation and normal function of β-cells, including their sensitivity to glucose (35), we found: in children under one year old, NeuroD1 was expressed in normal, single pancreatic cells; and most cells were located in the exocrine part, excretory ducts, or endotheliocytes. Previously, expression of this transcription factor in humans in non-tumor exocrine cells was described only in type 1 multiple endocrine neoplasia syndrome (36). In our study of CHI, NeuroD1 was expressed in both the endocrine and exocrine pancreas. It was also expressed in the walls of blood vessels and excretory ducts. The same immunohistochemical reaction is observed when dopa decarboxylase is expressed in the pancreas (37). The latter antigen in CHI is expressed in the exocrine and endocrine parts. In the normal pancreas, its expression level in the exocrine part is several times higher than in the islets of Langerhans. In our study, a similar situation was observed when analyzing the expression of dopamine receptors. In particular, DR2 was expressed both in the endocrine and exocrine pancreas, while DR5 was expressed only in the cytoplasm of blood vessel endothelial cells. In some patients, DR5 was also expressed in the cytoplasm of the excretory ducts.

Two possible mechanisms by which dopamine can influence insulin secretion have been described, central and local. The first mechanism is supported by the fact that dopamine is a precursor of norepinephrine and adrenaline and acts as a transmitter for the central and peripheral nervous system. It is known that the sympathetic nervous system inhibits insulin secretion with the help of norepinephrine and adrenaline, while acetylcholine released by parasympathetic neurons has the opposite effect (38). This process is regulated by glucose-sensitive neurons in the hypothalamus and hindbrain (39, 40). At the same time, it is known that NeuroD1 promotes the differentiation of adenohypophysis cells into corticotrophs expressing adrenocorticotropic hormone (41).

The local mechanism relates to monoamines of the pancreas. There are two types of monoamine oxidases: MAO-A and MAO-B. These two enzymes differ in their function. Substrates for MAO-A are epinephrine, norepinephrine, serotonin, histamine, dopamine, as well as many phenylethylamine and tryptamine psychoactive substances. Phenylethylamine and dopamine are also MAO-B substrates. The wide distribution of MAO-A has been proven in the exocrine part of the pancreas, while MAO-B is found only in centroacinar cells and epithelial cells of the ducts. In the endocrine part of the pancreas, MAO-A was detected in about 50% of cells, while MAO-B was found in a smaller number of cells and along the periphery of the islets (42). Since pancreatic cells accumulate monoamines (43, 44), catecholamines also play a role in the local mechanism of β-cell control. Insulin-secreting pancreatic β-cells perform dopamine synthesis and catabolism, as well as expressed all five subtypes of dopamine receptors. Dopamine negative regulation of glucose-stimulated insulin secretion is mediated by D2-like receptors including D2 (DR2) and D3 (DR3) receptors. Farino et al. (45) reported that uptake of L-DOPA is essential for establishing intracellular dopamine stores in β-cells. In normal circumstances, glucose stimulation significantly enhances L-DOPA uptake, leading to increased dopamine release and reduction of insulin secretion. Moreover, DR2 and DR3 together mediate dopaminergic inhibition of glucose-stimulated insulin secretion. The authors suggested that peripheral DR2 and DR3 receptors may play important roles in metabolism through the mechanism of inhibition of glucose-stimulated insulin secretion.

Same endocrine cell colocalization of insulin and dopamine has been demonstrated using electron microscopic autoradiography (46). Our study confirmed the joint localization of insulin and DR2 receptors in β-cells observed by Bini et al. (47). In addition, DR2-positive endocrine cells in patients with CHI-D were statistically significantly higher than: in the control (p <0.01); in CHI-F (in the node of adenomatous hyperplasia and outside of it); or in CHI-A (p < 0.05).

In our study, fluorescence microscopy confirmed the colocalization of DR2 receptors with NeuroD1 in the same endocrine and exocrine cells. However, the role of NeuroD1 as a transcriptional repressor and activator in the same endocrine cell cannot be underestimated. Moreover, as described by Itkin-Ansari et al. (48) and Chao et al. (49), this applies not only to β-, α-, and δ-cells, but also to PP cells. In addition, it has been proven that overexpression of NeuroD1, together with PDX1 and MAF BZIP transcription factor A (MafA), converts hepatocytes and intestinal cells into insulin-secreting cells (50–52). Possibly, the observed overexpression of NeuroD1 in the exocrine part, in all forms of CHI, indicates that this transcription factor can convert exocrine cells into endocrine ones with subsequent insulin secretion. In such a case, creation of a NeuroD1 inhibitor could aid in the treatment of CHI-A or CHI-D when partial pancreatectomy isn’t effective.

Thus, it seems that the function of NeuroD1 is not limited to participation in the formation and maintenance of β-cells. Its role in the formation and functioning of the exocrine part of the pancreas with excretory ducts is quite obvious. Apparently, this transcription factor is also involved in the central and local mechanisms of insulin secretion regulation.

Generally, SST should suppress insulin secretion. Our study showed that the relative numbers of cells with SST expression in CHI-F and CHI-D were significantly lower than in the control group. Perhaps a deficiency or biochemical “inferiority” of SST is another mechanism leading to the disease. Our opinion is confirmed by the work of Li et al. (53), which showed that ablation of SST-secreting cells in mice leads to neonatal death and dysregulation of basal synthesis and release of insulin by the pancreas. The authors proved that deficiency of δ-cells leads to excessive release of insulin with subsequent hypoglycemia.

In CHI, SSTR2 was expressed in 97% of patients, while SSTR5 was expressed 1.5 times less frequently in cases; this confirms the opinion of some authors about the functional dominance of SSTR2 in human endocrine cells (54). The use of SST analogues (both short-term and long-term) is a form of CHI drug therapy in which partial or subtotal pancreatectomy can be avoided in these patients. Octreotide inhibits insulin secretion by binding to SSTR2 and SSTR5. SSTR5 activation decreases the activity of the insulin gene promoter and inhibits calcium mobilization and acetylcholine activity. SST also suppresses the ATP-sensitive potassium channel (KATP channels), resulting in decreased insulin secretion. In a large series of studies, octreotide was found to be a safe and effective treatment for CHI patients not responding to diazoxide (22). At the same time, in one of our patients with CHI-D, SSTR2 and SSTR5 were not expressed. In one patient with CHI-A, SSTR5 was not expressed, and SSTR2 was not expressed in only 9% of cells.

We also, like Sempoux et al. (55) and Rahier et al. (56), confirm that CHI-D and CHI-F can be differentiated by morphological criteria even on frozen sections during surgery. Two approaches can be used: calculation of the relative number of large nuclei in a frozen section or, even faster, calculation of the absolute number of cells featuring nucleomegaly in smears. This was shown by a comparative analysis of the absolute and relative numbers of large nuclei in the field of view in different disease forms. Final differential diagnosis is carried out with an immunohistochemical study of paraffin sections. Any one of the main antibodies can be used with the same degree of reliability: insulin, Isl1, Nkx2.2, or chromogranin A.

Nevertheless, the most urgent problem is intraoperative localization of β-cell adenomatous structures. Winer et al. (57) have proposed intravenous administration of methylene blue (a near-infrared fluorophore) for intraoperative insulin imaging. The authors used the FLARE™ imaging system to acquire color video and near-infrared fluorescence images. Later, other suggestions were presented: Wada et al. (58) – near-infrared fluorophores T700-F and T700-H; Park et al. (59) – Ox61 for intraoperative imaging of neuroendocrine pancreatic tumors; and Boss et al. (60) – near-infrared fluorophore exendin-4-IRDye 800CW for insulin and CHI. In our opinion, the development and implementation of clinical neuroendocrine intraoperative navigation can not only completely cure a child with CHI-F (and in some cases CHI-A), but also improve the quality of postoperative life for CHI-D patients.

## Conclusion

For intraoperative differential diagnosis of CHI forms, counting the number of enlarged nuclei in pancreatic smears can be used as an additional verification of standard methods. If there are 7-10 cells with nucleomegaly in 10 fields of view, CHI-D is diagnosed. If 12-20 are found, CHI-F is established. The presence of such a cytological picture in all sent pancreatic fragments confirms CHI-D; presence in only some samples establishes CHI-F. With frozen pancreas sections, one can only focus on the relative number of endocrinocytes with nucleomegaly.

The most specific CHI markers, in general, were found to be NeuroD1 and Isl1. Insulin, Nkx2.2, chromogranin A, and SST can also be used for differential diagnosis of disease forms on paraffin sections. The number of cells expressing SST is reduced in CHI-F and CHI-D.

DR2 can also be used for morphological differential diagnosis of forms and for the diagnosis of CHI in general. The localization and intensity of dopamine receptor expression confirms the validity of the use of Pancreatic [18F] Fluoro-l-Dihydroxyphenylalanine (DOPA) Positron Emission Tomography.

Immunohistochemical study showed that DR2, as well as NeuroD1, are expressed not only in the endocrine pancreas, but also in the exocrine pancreas. Fluorescence microscopy revealed same-cell DR2/NeuroD1 colocalization. The latter fact may indicate participation of this transcription factor in the dopamine pathway. At the same time, creation of a NeuroD1 inhibitor would be advisable for targeted therapy of CHI-D or CHI-A when partial pancreatectomy is not effective. Our study also showed that CHI-A is distinguished by: the highest relative number of insulin-expressing endocrinocytes in the visual field; and the presence of a greater number of cells with large nuclei expressing the transcription factors Isl1 and Nkx2.2. This can potentially assist in differential diagnosis of CHI forms. In addition, CHI-A can be characterized by minimal changes in the pancreas with single large nuclei. This form causes diagnostic difficulties not only with Pancreatic [18F] Fluoro-l-Dihydroxyphenylalanine (DOPA) Positron Emission Tomography, but also with morphological analysis.

## Data Availability Statement

The original contributions presented in the study are included in the article/supplementary material. Further inquiries can be directed to the corresponding author.

## Ethics Statement

The studies involving human participants were reviewed and approved by Ethics Committee of the Almazov National Medical Research Centre. Written informed consent for participation was not required for this study in accordance with the national legislation and the institutional requirements.

## Author Contributions

LM, VB, and IN contributed to conception and design of the study. AP, DR, and AS organized the database. AP performed the statistical analysis. LM wrote the first draft of the manuscript. DR and AP wrote sections of the manuscript. All authors contributed to the article and approved the submitted version.

## Conflict of Interest

The authors declare that the research was conducted in the absence of any commercial or financial relationships that could be construed as a potential conflict of interest.

## Publisher’s Note

All claims expressed in this article are solely those of the authors and do not necessarily represent those of their affiliated organizations, or those of the publisher, the editors and the reviewers. Any product that may be evaluated in this article, or claim that may be made by its manufacturer, is not guaranteed or endorsed by the publisher.
